# Orbital-Resolved DFT*+U* for Molecules and Solids

**DOI:** 10.1021/acs.jctc.3c01403

**Published:** 2024-05-31

**Authors:** Eric Macke, Iurii Timrov, Nicola Marzari, Lucio Colombi Ciacchi

**Affiliations:** † Faculty of Production Engineering, Bremen Center for Computational Materials Science and MAPEX Center for Materials and Processes, Hybrid Materials Interfaces Group, University of Bremen, Am Fallturm 1, 28359 Bremen, Germany; ‡ Theory and Simulation of Materials (THEOS) and National Centre for Computational Design and Discovery of Novel Materials (MARVEL), École Polytechnique Fédérale de Lausanne, CH-1015 Lausanne, Switzerland; ¶ University of Bremen Excellence Chair, Bremen Center for Computational Materials Science, Am Fallturm 1, 28359 Bremen, Germany

## Abstract

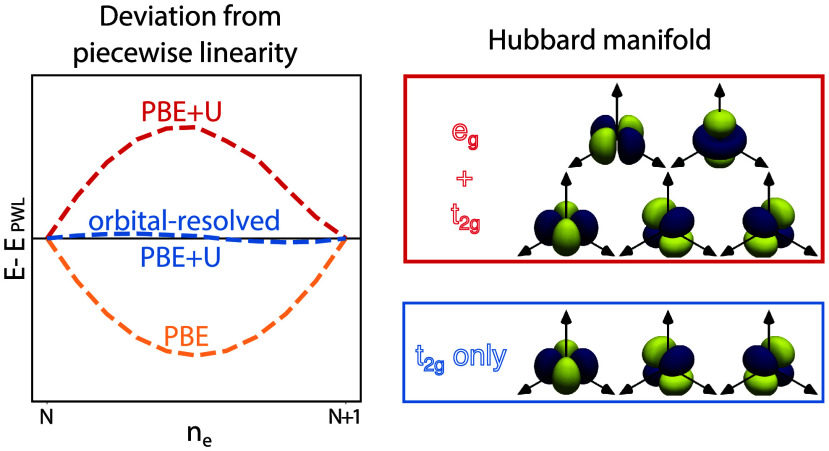

We present an orbital-resolved extension of the Hubbard *U* correction to density-functional theory (DFT). Compared to the conventional shell-averaged approach, the prediction of energetic, electronic and structural properties is strongly improved, particularly for compounds characterized by both localized and hybridized states in the Hubbard manifold. The numerical values of all Hubbard parameters are readily obtained from linear-response calculations. The relevance of this more refined approach is showcased by its application to bulk solids pyrite (FeS_2_) and pyrolusite (β-MnO_2_), as well as to six Fe(II) molecular complexes. Our findings indicate that a careful definition of Hubbard manifolds is indispensable for extending the applicability of DFT+*U* beyond its current boundaries. The present orbital-resolved scheme aims to provide a computationally undemanding yet accurate tool for electronic structure calculations of charge-transfer insulators, transition-metal (TM) complexes and other compounds displaying significant orbital hybridization.

## 1 Introduction

Hubbard corrections are among the most widely used improvements to approximate Kohn–Sham (KS) density-functional theory (DFT).^1,2^ The combination of DFT with the Hubbard model,^3^ referred to as DFT+*U*, was inspired by this widely studied model of electron correlations and introduced to improve the description provided by local or semilocal exchange-correlation (xc) functionals, such as the local-density approximation (LDA) and the generalized-gradient approximation (GGA), for the case of Mott-Hubbard insulators^4−6^ or more broadly strongly correlated electrons.

It was recognized early on^7,8^ that a simplified, rotationally invariant formulation of DFT+*U*^9^ provided a natural connection to the requirement of piecewise linearity of the exact energy functional:^10,11^ the Hubbard correction of Dudarev et al. effectively removes the nonlinear (almost quadratic) behavior of the total energy with respect to the occupation of the Hubbard manifold, and replaces it with a linear term. In this light, the strength of the *U* parameter can be determined fully from first-principles (for practical reasons using the response of the occupations to a linear perturbation^7,8^), so that the quadratic curvature is removed by DFT+*U*. This connection is heuristic, and relies on the (very reasonable) assumption that the localized electrons in the *d* or *f* manifold are only weakly interacting with the rest of the electron bath, so that this manifold also follows the condition of piecewise linearity (PWL) that is in principle valid only for the total energy functional.^10^ The overstabilization of fractional occupations in standard (semi)local functionals is driven by the incomplete cancellation from the xc functional to the Hartree term (cancellation that is exact instead in Hartree–Fock), leading to a strong (one-electron) self-interaction component. This is particularly severe for localized electrons, that overdelocalize and overhybridize with their ligands. So, the DFT+*U* correction should be seen as a self-interaction correction, providing an approximate screened Fock-like contribution; this was stated early on, highlighting how even for molecular systems containing only one site (i.e., one TM atom) the electronic-structure description provided by DFT+*U* is greatly improved, both qualitatively and quantitatively.^12^ In other words, while the original Hubbard model was concerned with the correlation effects that appear on a lattice, where each site can only contain 0, 1, or 2 electrons, the functional form that the model inspires, once applied to the continuum of the electron gas of approximate DFT, does not any more account for correlations between sites, but counteracts the tendency of strongly localized electrons to hybridize with their ligands, driven by incomplete cancellation in the Hartree term of the one-electron self-interaction. This can be easily argued not only by applying DFT+*U* to molecules,^12^ but also by the simple observation that changing the value of *U* changes the charge transfer to/from the ligands,^13^ but does not transfer charge to the Hubbard manifolds on the other sites, highlighting the completely different physics of the Hubbard Hamiltonian on a lattice, and the Hubbard *U* correction in the continuum of KS electrons. Incidentally, strongly correlated materials have almost invariably very localized *d* or *f* electrons, so do benefit from DFT+*U* – but not because the latter improves the treatment of correlations, but just because it decreases the self-interaction errors (SIE). It is important to iterate that PWL is a property that a system must obey on the *global* scale, i.e., with respect to the changes in the total number of electrons.^14^ DFT+*U*, on the other hand, seeks to eliminate the curvature *locally* by restoring the piecewise linear behavior in a subspace known as the Hubbard manifold.^8,15^ The relation between self-interaction and PWL in many-electron systems is somewhat confusing; in fact, Koopmans functionals^16,17^ impose (very accurately) PWL on each orbital in a given system, but do not correct self-interaction; the integer Koopmans functional (KI) provides the same exact total energy of the base functional (LDA or GGA), but a completely different spectrum. Other recent approaches (based on DFT+*U*)^18−21^ even aim to fulfill the flat-plane condition,^22^ which, in addition to PWL, demands energetic invariance with respect to changes in electron spin in half filled orbitals.

A key challenge in DFT+*U* arises from the (*a priori*) choice of the value of the on-site Hubbard *U* parameter. Often, *U* is considered a tunable quantity, adjusted to achieve agreement with experimental results for specific properties of interest such as band gaps, lattice parameters, magnetic moments, or formation enthalpies.^23−26^ However, this empirical procedure possesses limited predictive capabilities due to its reliance on experimental data; not to mention that the KS levels used to compute band gaps are incorrect even in exact DFT (bar the HOMO). Alternatively, *U* values can be computed from first principles. The different methods that have been proposed for this purpose can be classified into three groups: the constrained DFT approach (cDFT),^27−35^ the constrained random phase approximation (cRPA),^36−40^ and Hartree–Fock based approaches.^41−46^ The linear-response formulation of constrained DFT (LR-cDFT)^8^ has become a method of choice for many DFT+*U* studies.^47−55^ A recent reformulation of this method in terms of density-functional perturbation theory (DFPT)^56,57^ has significantly enhanced its success. This reformulation enables the replacement of computationally expensive supercells with primitive unit cells, utilizing monochromatic perturbations. As a result, the computational burden of determining Hubbard parameters is substantially reduced.

As evident from the large variety of available methods, considerable attention has been devoted to the numerical evaluation of *U* for a given manifold. However, the question how this manifold must be defined, that is, *which* electronic states require on-site Hubbard corrections, is a critical yet often overlooked aspect in DFT+*U*. Recalling that the main motivation of Hubbard *U* corrections lies in the mitigation of local SIE through recovery of PWL of the total energy, the Hubbard manifold should contain those and only those states that substantially contribute to the former. Oftentimes, self-interaction occurs in partially occupied *d* and *f* shells due to their high electron count and localization; hence, these are the traditional targets of Hubbard *U* corrections. Nevertheless, self-interaction can also manifest itself in *s* and *p* shells, and even in localized molecular orbitals (MO).^58^ Moreover, the definition of the Hubbard manifold also involves choosing so-called Hubbard-projector functions that suitably represent the states to be corrected (details follow in Section 2.2). In fact, it has been shown that the choice of the projector function can have a strong impact on the calculation, especially when orbital overlap is appreciable.^50,59−61^ Therefore, it is essential to choose a *U* value that is consistent with the projectors used (see appendix of ref (62)).

The significance of judiciously selecting the Hubbard manifold becomes particularly evident in the context of optical properties. Although DFT is a total energy theory and KS orbitals have no direct physical meaning except for the highest occupied molecular orbital (HOMO) in exact DFT, KS eigenvalues are commonly used as estimates for experimental quasiparticle energies. Unfortunately, uncorrected (semi)local DFT functionals tend to severely underestimate properties such as band gaps and ionization energies; a fact that has been linked to deviations from PWL.^16,17,63^ Thus, Koopmans-compliant functionals^16,17,64^ systematically improve spectral properties by imposing PWL on all orbitals in the system. For example, a study by Nguyen et al. found an average band gap error of only 0.22 eV for a test set of 30 semiconductors.^65^ Conversely, DFT+*U* can only achieve such improvements if the Hubbard manifold sufficiently overlaps with the frontier orbitals.^15,50,66^ This might explain why DFT+*U* has been successfully applied to many Mott-Hubbard insulators, while its predictive capabilities are less reliable when applied to charge-transfer insulators. While in the former both the valence band maximum (VBM) and the conduction band minimum (CBM) primarily consist of localized TM *d* states, the frontier states of the latter possess significant ligand orbital character. In this case, the sole correction of TM *d* shells does not necessarily target all major sources of SIE. Additionally, the representation of the hybridized orbitals through atomic-like Hubbard projectors can be deceptive.

Some authors have explored including ligand orbitals in the on-site Hubbard manifold, for example applying *U* corrections to O-*p* and S-*p* shells of TM compounds.^50,53,67−69^ The extended DFT+*U*+*V* framework^58^ was adopted for scenarios where hybridization plays an eminent role. This method augments DFT+*U* by an intersite Hubbard *V* term, and so acts on combinations of projectors located on different sites, enhancing accuracy and transferability.^46,59,70−79^ Finally, many open-shell systems require unlike-spin terms such as Hund’s *J*. In DFT+*U*+*J*, these terms introduce an unlike-spin exchange correction, while also reducing the effective value of the *U* parameter.^53,66,68,80,81^ It remains to be shown in how far a single-determinant method such as DFT+*U*(+*J*) suffices to adequately describe compounds with a strong multireference character, but it is likely that Hubbard *U* and Hund’s *J* corrections can improve the description of approximate single-determinant solutions to multiconfigurational systems^8,82^ (often obtained by breaking the symmetry^83^).

What is striking about the aforementioned approaches is that the *d* shell Hubbard manifold is either *extended* by additional states (*U* on ligands or *V* on molecular orbitals) or internally *rebalanced* (DFT+*U*+*J*). But what if the practice of including the entire *d* shell is, in itself, problematic? Recent work by Mariano et al. suggests that this might indeed occur in some cases.^84,85^ Their study revealed that applying *U* corrections to the *d* shells of Fe in strong-field Fe(II) hexacomplexes leads to a spurious suppression of low-spin states due to what appears to be an overcorrection of hybridized *e*_*g*_ orbitals. Furthermore, the use of *ab initio* Hubbard *U* parameters derived from LR-cDFT led to a significant decrease in the overall accuracy of the spin-state energies when compared to empirical *U* values. These and other observations suggest a critical analysis of the current practice of how (shell-averaged) Hubbard *U* corrections are applied to DFT. After all, the correction of all magnetic quantum orbitals within a given shell using the same scalar *U* parameter is inherently a simplistic approximation.^86^

Therefore, it is worthwhile to investigate whether departing from the shell-averaged approximation can improve DFT+*U* calculations concerning energetic, structural, and magnetic properties. This possibility has been explored in a few works. For example, Solovyev et al. showed that a selective application of *U* corrections to the *t*_2*g*_ manifolds of La*M*O_3_ perovskites (*M*=Ti–Cu) significantly improves the agreement of band gaps and magnetic orderings with experiments compared to both uncorrected LDA and shell-averaged LDA+*U*.^87^ The authors argue that *e*_*g*_ electrons are reasonably described by the uncorrected LDA functional because of their “itinerant” behavior that arises due to the strong σ overlap between O-2*p* and M-*e*_*g*_ orbitals. Pickett et al. adopted the cDFT approach to compute the Hubbard parameters of Fe in bulk FeO as a matrix (**U**) with *U* values specific to the *t*_2*g*_ and *e*_*g*_ manifolds, respectively.^32^ Using a different scheme, orbital-resolved Hubbard parameters were obtained by mapping shell-averaged *U* and *J* parameters onto orbital-dependent interaction and exchange matrices *U*_*mm*′_ and *J*_*mm*′_ using atomic Slater integrals and Gaunt’s numbers.^88−91^ Other works focused on the spin-dependence of *U*, finding that the spin-resolved on-site parameters can be pivotal for a physical description of magnetic systems.^54,55,92^

Despite these early efforts, orbital-resolved Hubbard *U* parameters have not gained widespread use in the DFT community. This is surprising in light of the fact that orbital-specific Hubbard manifolds are quite common in the dynamical mean-field theory (DMFT) community.^93−96^ In the latter, Hubbard *U* parameters are routinely computed for manifolds that range from groups of orbitals (e.g., *t*_2*g*_ models) to combinations of multiple shells localized on different atoms (e.g., *d* – *p* models)^94^ using cRPA in conjunction with various sets of Wannier projector functions that encompass the specific manifold of interest.^36,37^ To date, only a few publicly available DFT codes incorporate this capability. For example, DFT codes that use Wannier function-based Hubbard projectors (e.g., ONETEP(97)) indirectly facilitate orbital-resolved DFT+*U* calculations as the Wannier functions can be chosen to only represent the desired subset of orbitals, e.g., *t*_2*g*_.^61^

In this paper we present a user-friendly yet general implementation of orbital-resolved DFT+*U* that works with any kind of Hubbard projector. The numerical values of the necessary parameters are extracted from first-principles using an orbital-resolved LR-cDFT approach.^8^ We benchmark the orbital-resolved scheme by carrying out calculations of bulk pyrite (FeS_2_), bulk pyrolusite (β – MnO_2_) and six Fe(II) molecular hexacomplexes of varying ligand strength. For all of these strongly covalent compounds, the refined approach leads to a substantial improvement in the prediction of structural and energetic properties, aligning more closely with experimental observations than conventional DFT+*U* or even DFT+*U*+*V*. Moreover, we observe that the orbital-resolved *U* parameters are considerably smaller, by up to 80%, than their corresponding shell-averaged counterparts.

The remainder of the paper is organized as follows. In Section 2, we describe the transition from the customary, shell-averaged implementations of the DFT+*U* energy functional and the LR-cDFT approach to their generalized, orbital-resolved forms. Subsequently, Section 3 briefly lists the relevant technical details of the calculations, whose results are presented and discussed in Section 4. Section 5 is dedicated to the relationship between the orbital-resolved (on-site) Hubbard *U* presented in this work and the (intersite) *V* terms of ref (58). Finally, we summarize the main conclusions in Section 6.

## 2 DFT+*U*: Orbital-Resolved Hubbard Parameters

### 2.1 The Energy Functional

As this work focuses on the on-site *U* term of Hubbard-corrected DFT, we start from the widespread shell-averaged and rotationally invariant formulation of DFT+*U* by Dudarev et al.^9^ Written in a way that allows for the simultaneous correction of multiple subshells on the same atom, the energy functional reads:^98^
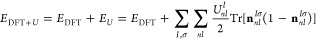
1where *E*_DFT_ is the DFT total energy computed with standard (semi)local xc functionals, *U*_*nl*_^*I*^ is an effective on-site Hubbard parameter, and **n**_*nl*_^*Iσ*^ is the orbital occupation matrix. The summation over the principal (*n*) and orbital (*l*) quantum numbers implies that Hubbard *U* corrections can be applied simultaneously to multiple subshells of the same atom,^50,58^ although it usually suffices to treat the valence shell alone. For the sake of clarity, we henceforth omit the index *nl*, assuming that the Hubbard manifolds consist of, at most, one subshell per atom. The orbital occupation matrix defines how the Hubbard correction is applied. For a given spin σ, its elements are computed by projecting the valence KS wave functions onto the Hubbard manifold of an atom *I*:
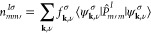
2where *m* and *m*′ are the magnetic quantum numbers of the Hubbard manifold, ν represents the band labels of the KS wave functions, **k** indicates points in the first Brillouin zone, and *f*_**k**,ν_^σ^ are occupations of the KS wave functions ψ_**k**,ν_^σ^. If atomic-like orbitals (φ_*m*_^*I*^) are used as projector functions (*vide infra*), one may define . Note, however, that if plane-wave basis sets are used, the expression of *P̂* also depends on the type of the pseudopotential.^57^ Applying Hubbard *U* corrections also modifies the KS potential of the target orbitals according to
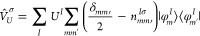
3where δ_*mm*′_ is the Kronecker delta. It is evident from eq 3 that Hubbard *U* corrections exert a stabilizing influence on fully occupied orbitals, reducing their KS potential by up to *U*/2, while producing the opposite effect on empty orbitals.

Provided a correct normalization of the Hubbard projector functions, the eigenvalues of  express the occupation of the 2*l* + 1 orbitals with numbers between 0 (fully empty) and 1 (fully occupied). They are obtained by solving the eigenvalue problem^8^
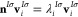
4where λ_*i*_^*Iσ*^ and **v**_*i*_^*Iσ*^ are the eigenvalues and eigenvectors, respectively, and *i* is a dummy index running from 1 to 2*l* + 1. This allows us to rewrite the *E*_*U*_ contribution of eq 1 more concisely in terms of these eigenvalues:
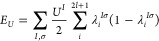
5

Within this diagonal representation, eq 5 can be readily generalized to become an orbital-resolved correction:
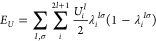
6

Here, *U*_*i*_^*I*^ is now an effective on-site Hubbard parameter specific to the *i*th orbital of the Hubbard manifold localized on atom *I*. Similar generalizations of the shell-averaged DFT+*U* functional were already postulated by Pickett et al. and used by Solovyev et al., although these early works adopted DFT+*U* energy functionals different from Dudarev et al.’s formulation.^32,87^

In practical calculations, not every orbital requires a distinct *U*_*i*_^*I*^, and it might suffice to distinguish between the irreducible representations that follow from local point group symmetry. For instance, considering the *d* shell of an octahedrally coordinated atom with local *O*_*h*_ point group symmetry, eq 6 can be written as
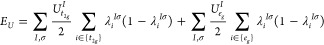
7where  and  are the Hubbard parameters for the *t*_2*g*_ and *e*_*g*_ orbitals, respectively, and *i* ∈ {*t*_2*g*_} means that *i* runs over the orbital indices of the *t*_2*g*_ subshell, while *i* ∈ {*e*_*g*_} means that *i* runs over the orbital indices of the *e*_*g*_ subshell. It is also possible to selectively exclude specific orbitals from receiving Hubbard corrections by setting *U*_*i*_^*I*^=0.

The calculation of the Hubbard potential (eq 3) is also performed in the diagonal representation by rotating the atomic-like orbitals φ_*m*_^*I*^ from the global to the local coordinate system using the eigenvectors **v**_*i*_^*Iσ*^. However, after computing the orbital-resolved contributions to the Hubbard potential , we perform a backrotation into the nondiagonal representation as this allows to use existing implementations of density mixing as well as the calculation of forces and stresses with no further adaption.

A crucial aspect for a successful application of Hubbard corrections lies in finding a suitable projector function for the target manifold, as this controls the occupation eigenvalues λ_*i*_^*Iσ*^ (eq 2) which govern the corrective Hubbard energy. The next subsection offers a concise introduction to the prevailing and commonly utilized approaches.

### 2.2 Hubbard Projector Functions

There are many ways to define Hubbard projector functions within the DFT+*U* approach, and the reader is referred to ref (99) and references therein for a more comprehensive overview. For electronic-structure codes employing a localized basis set, natural choices for Hubbard occupations are either Mulliken or Löwdin population matrices.^100^ In contrast, codes based on plane-wave basis sets often use atomic orbitals as projectors,^8,101^ whose localized functions are parametrized with free-atom calculations and then stored in the pseudopotentials. During the generation of pseudopotentials, the atomic orbitals are chosen to be orthonormal to all other orbitals centered on the same atom. However, this cannot guarantee orthogonality to orbitals localized on other sites during practical calculations. Hence, these projector functions are referred to as nonorthogonalized atomic orbitals (NAO).^59^ While straightforward to implement and use, NAO may display spatially extended “tails”, potentially resulting in the same domain being tackled twice by Hubbard corrections if the atomic orbitals of two neighboring atoms overlap.

Truncation spheres^101^ provide a means of ruling out such double counting by cutting off possible tails.^1^ However, the radius of these spheres represents an additional parameter that affects the obtained occupation numbers.^102^ The value of this parameter varies depending on the code utilized and may require manual adjustment (e.g., muffin-tin radius in the linearized augmented plane-wave approach) or can be embedded within the pseudopotential (typical of projector-augmented-wave (PAW) pseudopotentials).

An alternative, parameter-free approach to circumvent truncation involves orthogonalizing NAO across all atomic sites, e.g., using Löwdin’s scheme,^103^ thus transforming them into orthogonalized atomic orbitals (OAO).^59,99^ The resulting intersite orthogonality clears the overlap of projectors on different sites^99^ and even accounts for possible hybridization between them, albeit to a limited extent. Several benchmark studies have shown that OAO projectors consistently outperform NAO projectors with respect to structural, electronic and spectral properties.^50,59,75^

Finally, Wannier functions are also viable Hubbard projector functions.^34,104,105^ Specifically, maximally localized Wannier functions (MLWF)^106,107^ can separate manifolds in a system-specific fashion,^108^ and can serve as effective Hubbard projector functions within the generalized DFT+*U* framework presented in eq 6. Despite the extensive use of Wannier functions, their current adoption in DFT+*U* calculations remains limited,^61,109,110^ possibly due to the nontrivial steps of finding an appropriate starting guess and disentangling overlapping bands during wannierization. Moreover, features relevant for practical studies such as the calculation of forces and stresses are cumbersome to implement.

### 2.3 LR-cDFT to Compute Orbital-Resolved *U* Parameters

LR-cDFT is based on using the DFT+*U* energy functional to (heuristically) restore PWL for the Hubbard manifold in (semi)local DFT functionals suffering from electron self-interaction.^8,12^ An important manifestation of the latter are so-called fractional charge errors (FCE), which are spurious (usually convex) deviations from linearity of *E*_DFT_ with respect to fractional addition or removal of charge,^15^ i.e.,
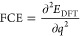
8where *q* represents the charge of the system under consideration. Inspection of eq 1 shows that the Hubbard *U* correction amounts to removing a quadratic term and adding a linear one, scaled by the numerical value of *U*. Note, however, that the curvature removed by eq 1 is not with respect to the total charge of the system *q* but instead with respect to the projected *local* occupation of the Hubbard manifold **n**^*Iσ*^. Thus, a fundamental assumption is that the electrons in this localized Hubbard manifold are the most affected by self-interaction and can be dealt with separately. This interpretation makes it possible to define the value of Hubbard *U* as the one for which the second derivative of the total energy functional becomes zero with respect to changes in the occupation of the shell,^8^
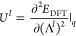
9where we now use  to define the local occupation of the Hubbard manifold. Because a direct control of orbital occupations is not tractable in codes that obtain them as output quantities, Lagrange multipliers α are introduced to linearly shift the potential of the Hubbard manifold and thus indirectly control Λ^*I*^ (see refs (7, 8, 32) for the derivation). Then, two response matrices are defined using finite differences:
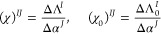
10where χ represents the self-consistent, screened response of the manifold, whereas χ_0_ is due to the noninteracting, unscreened response coming from the rehybridization of the electronic states that results from the perturbation. In many plane-wave codes, these latter noninteracting orbital occupations Λ_0_^*I*^ can be obtained from the first iteration in the self-consistent cycle of the perturbative calculation. Since the response χ_0_ is unrelated to electron self-interaction, it must be subtracted from the interacting (i.e., screened) response when computing *U*:
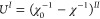
11

In practice, the response functions are obtained by either applying multiple small (positive and negative) perturbations to the shells of interest of a converged ground state^8^ (with periodic systems requiring a supercell approach to avoid interactions between a perturbed Hubbard manifold and its periodic images) or from DFPT^56,57,111^ using the response to monochromatic perturbations in a primitive cell. Importantly, during peturbative calculations, the Hubbard potential  should be fixed to its self-consistent *unperturbed* DFT+*U* value, so as to ensure that *U* is evaluated using only the DFT-part of the curvature.^47,58,112^

Orbital-resolved Hubbard *U* parameters can be evaluated using the formalism of eqs 10 and 11 by adapting the definition of the total occupation of the Hubbard manifold. In the most general case, every magnetic quantum orbital *i* of the *nl* subshell can acquire an individual Hubbard parameter. The occupation of such a manifold is given by
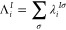
12

With this, the elements of the response matrices (eq 10) can be redefined as
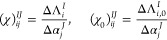
13while the expression for the orbital-resolved on-site Hubbard *U* parameters becomes
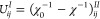
14

The generalized formalism of eq 14 not only accommodates orbital-resolved on-site *U* parameters, but allows for the determination of intersite parameters (*U*_*ij*_^*IJ*^ with *I* ≠ *J*, or, following the nomenclature of DFT+*U*+*V*, *V*_*ij*_^*IJ*^), as well as on-site interorbital parameters (*U*_*ij*_^*II*^ for *i* ≠ *j*). Nevertheless, the primary emphasis of this study lies in investigating the on-site intramanifold parameters *U*_*ii*_^*II*^ ≡ *U*_*i*_^*I*^.

For the sake of illustration, let us consider a *d* shell of an atom exhibiting local *O*_*h*_ symmetry. Also, we assume that this atom be the only Hubbard atom in the system, thereby enabling us to neglect intersite responses and to drop the superscript *I*. Then, one can define an orbital-resolved Hubbard matrix of size 2 × 2 that reads
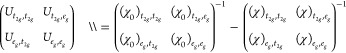
15where the indices represent the response in occupations of manifold *a* to perturbation of manifold *b*, , for instance . Pickett et al. showed that the orbital-resolved matrix elements of eq 15 are related to the shell-averaged Hubbard parameter *U* through a sum rule.^32^ A slightly adapted form of this rule that accounts for the role of the noninteracting response reads^2^
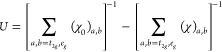
16

The lesson to learn from eq 16 lies in the off-diagonal values of χ, whose physical implication consists in intrashell screening (in this example *e*_*g*_ ↔ *t*_2*g*_). Since the sign of these off-diagonal values is normally opposite to that of the diagonal ones,^3^ they diminish the contribution of the term *∑*_*ab*_(χ)_*a*,*b*_, which may substantially increase *U* values upon computation of the inverse. Note that χ_0_ is not affected by this, because the off-diagonal elements of the unscreened response are zero by definition (except for numerical noise). In the extreme scenario where perturbations are exclusively screened within the same shell, the sum over the off-diagonal elements equals the trace of χ, causing det(χ) to approach zero, leading to *U* → *∞*. Consequently, if the current definition of the response matrices is used to compute orbital-resolved *U* parameters, the determinant required for the full inversion of χ inadvertently reintroduces the shell-averaging of the response. This is evident in the findings of the aforementioned study by Pickett et al., where the computed *U*_*t*_2*g*__ (≡*U*_*t*_2*g*_,*t*_2*g*__) displayed only minimal differences compared to *U*_*e*_*g*__ (≡*U*_*e*_*g*_,*e*_*g*__) and the shell-averaged *U*.^32^ It should be noted that singularities in χ of shells with little to no alternative intershell screening channels occur regardless of the way the shell is partitioned. Even by replacing the *t*_2*g*_/*e*_*g*_ distinction by one where all five *d* orbitals are treated individually, the determinant would still be singular.

Therefore, in order to derive on-site *U* parameters that explicitly incorporate intrashell screening effects (and avoid singularities), it is necessary to set the off-diagonal matrix elements of χ and χ_0_ to zero before computing their inverses. This scheme was employed by Linscott et al., who refer to it as pointwise (“1 × 1”) inversion, in order to compute screened and spin-resolved Hubbard parameters for metal aquo complexes.^55^ Solovyev et al. also applied this strategy, albeit implicitly, to obtain screened *U* parameters specific to *t*_2*g*_ in perovskites.^87^ Although the removal of off-diagonal elements seems a drastic approximation, it allows for a more tailored definition of Hubbard manifolds compared to the conventional DFT+*U* approach. The latter assumes (without proof) that ligand orbitals account for the majority of screening while the role of intrashell interactions is neglected. In fact, several cRPA studies suggest that the opposite is true, showing that intrashell screening can be as significant, or even more so, as intershell screening.^94,95^ Although we are not aware of a metric that allows to gauge how much screening should be present during the evaluation of Hubbard parameters, the examples in Sec. 4 demonstrate that intrashell screening is crucial for some manifolds. That being the case, we expect DFT+*U* calculations to benefit from using *U* parameters whose numerical values reflect the true screening environment rather than values computed for a system where important screening channels are absent (either due to orbitals being perturbed simultaneously or due to their inadvertent elimination by performing a full inversion of the orbital-resolved χ matrix). An important caveat to the LR-cDFT approach that also affects the orbital-resolved form presented here is that unphysical *U* values may result when it is applied to fully occupied manifolds. This is a well-known limitation^113,114^ that follows from the fact that the response of a deep-lying state to a relatively small perturbation (≈0.05 eV) is often on the same order of magnitude as the numerical noise, leading to instabilities during the inversion of the response matrices.

## 3 Computational Details

All calculations are carried out using the Quantum ESPRESSO distribution.^115−117^ We have incorporated the capability to utilize and determine orbital-resolved Hubbard *U* parameters with the pw.x code and will make this feature available in the next official release. Structure and isosurface plots for pyrite (FeS_2_) and pyrolusite (β – MnO_2_) are generated using VESTA.^118^ Unless stated otherwise, all systems are structurally optimized using the Broyden-Fletcher-Goldfarb-Shanno (BFGS) algorithm^119^ with convergence thresholds of 10^–4^ Ry, 10^–3^ Ry/Bohr, and 0.5 kbar for the total energy, forces, and pressure, respectively. KS wave functions (charge density) are expanded in plane waves up to a kinetic-energy cutoff of 90 Ry (1080 Ry) using PBE^120^ pseudopotentials for pyrite and the Fe(II) molecular complexes, and PBEsol^154^ pseudopotentials^4^ for β – MnO_2_ taken from the SSSP Precision library v. 1.1.2.^124,125^ The projected density of states (PDOS) is obtained using a Gaussian smearing with a broadening parameter of 0.02 Ry and employing the *diag_basis* feature of the projwfc.x code, which projects the wave functions onto the eigenstates of the occupation matrix rather than using unrotated atomic orbital projectors.^59^ This allows for a clear distinction between *t*_*g*_/*t*_2*g*_ and *e*_*g*_ states, regardless of the orientation of the global coordinate system. Fe(II) molecular complexes are simulated at a fixed +2 charge state in cubic boxes with an edge length of 15 Å and using only the Γ point to sample the Brillouin zone. The total magnetization of the molecular complexes is always fixed to either 4.0 μ_B_ or 0.0 μ_B_ in order to compute high-spin (HS) and low-spin (LS) configurations, respectively. The starting geometries of the Fe(II) molecular complexes are taken from the SI of ref (84), whereas experimental structures are chosen as a starting points for FeS_2_ and β – MnO_2_. The Brillouin zones of FeS_2_ and β – MnO_2_ are sampled with uniform Γ-centered Monkhorst–Pack meshes of sizes 9 × 9 × 9 and 4 × 4 × 6, respectively.

We use Löwdin-orthogonalized atomic orbitals as Hubbard projectors (OAO)^99,103^ for all DFT+*U* calculations. Orbital-resolved Hubbard *U* parameters and orbital-resolved perturbations (α) are applied by targeting specific eigenstates of the occupation matrix (e.g., those corresponding to *t*_2*g*_ orbitals). A tracking algorithm^126^ implemented in the code ensures that the corrections are consistently applied to the desired eigenstates, even if the states’ eigenvalue indices shift during the self-consistent field (SCF) calculation (see SI for further details). As indicated in Section 2.1, the change in potential due to Hubbard *U* corrections or linear perturbations is computed within the diagonal representation but is rotated back to the nondiagonal one. This transformation is always performed using the eigenvectors of the *current* occupation matrix, i.e., the occupation matrix corresponding to the charge density of the current SCF iteration. This choice entails some inconsistency because the eigenvectors (of the occupation matrix) are altered by active Hubbard corrections or perturbations. In practice, however, these alterations are minimal and negligible (Table SI 1). Moreover, the self-consistency scheme explained below not only converges the Hubbard parameters but also the eigenvectors of the occupation matrix, thereby eliminating the inconsistency.

As the orbitals’ response depends on the system’s electronic structure, calculated Hubbard parameters may vary significantly when transitioning from a PBE/PBEsol ground state to a PBE+*U*/PBEsol+*U* one. Therefore, we achieve self-consistency of the computed *U* values by employing the iterative procedure detailed in section 4 of ref (57), which has also been used in previous works.^47,48,71^ In this scheme, one starts from an initial set of Hubbard parameters *U*_*in*_ and an initial structure (where *U*_*in*_ can be zero or can adopt a finite value based on an educated guess^35,57^). First, a geometry optimization of the structure is performed where energies, forces and stresses are evaluated using DFT+*U* with *U* = *U*_*in*_. The DFT+*U* ground state of the relaxed structure provides the starting point for perturbative calculations using either LR-cDFT or DFPT. Next, the perturbative calculations are carried out, yielding a new set of Hubbard parameters *U*_*out*_. If the difference between the input and the output parameters is smaller than a predefined threshold (in this work we use ∼0.1 eV), *U*_*in*_ and the structure are considered to be converged. Otherwise, *U*_*in*_ is set equal to *U*_*out*_ and the procedure is restarted from the structural relaxation step. For a detailed discussion of this and an alternative self-consistency scheme,^12^ the reader is referred to ref (127).

For the perturbative calculations, the DFPT implementation^56,57^ of the HP code (hp.x)^111^ included in Quantum ESPRESSO is employed to evaluate the shell-averaged Hubbard parameters. We use **q** point meshes of size 2 × 2 × 2 for FeS_2_ and β – MnO_2_, and 1 × 1 × 1 for the Fe(II) molecular complexes, respectively. Orbital-resolved *U* parameters are computed according to the LR-cDFT approach by applying perturbations of α = [−0.05, 0.05]eV to the manifold(s) of interest and recording the noninteracting and interacting responses of the orbital occupations. To avoid interactions of perturbations with their periodic images, the calculations of FeS_2_ and β – MnO_2_ are conducted in 2 × 2 × 2 supercells containing 96 atoms. We emphasize that DFPT and LR-cDFT are equivalent by construction, and therefore yield the same Hubbard parameters when applied to identical systems.^56^ The resulting Hubbard parameters are reported in Section 4.

## 4 Results and Discussion

### 4.1 Pyrite (FeS_2_)

#### 4.1.1 Challenging Theoretical Description

**Figure 1:**
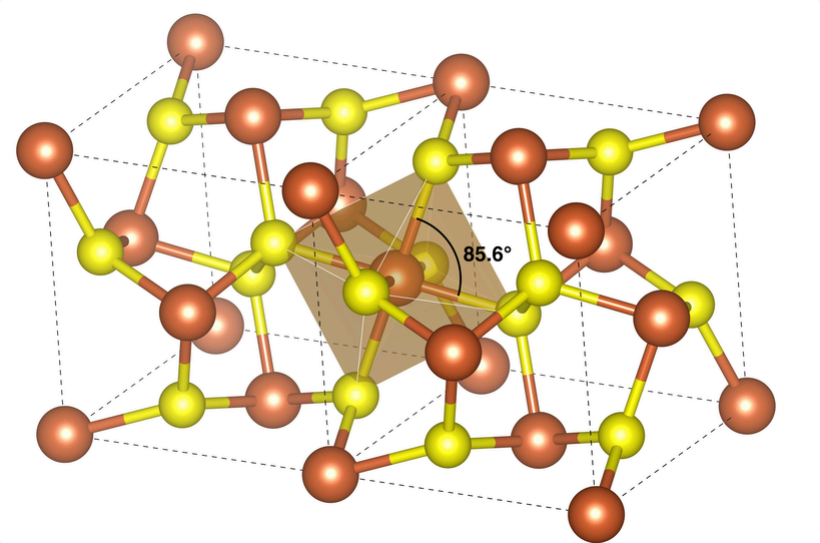
Experimental crystal structure of pyrite (FeS_2_). Fe (brown) is octrahedrally coordinated by S (yellow), which also forms characteristic S– S dimers. The octahedra are slightly distorted and display angles different from 90°.

Under normal conditions, pyrite is the stable polymorph of FeS_2_ and crystallizes in the cubic space group Pa3̅ with an experimental lattice parameter *a* = 5.418 Å.^128^ The crystal structure, shown in Figure 1, consists of S_2_ dimers octahedrally coordinating Fe^2+^ ions, which form a *fcc* sublattice.^129^ At 0 K, the compound is diamagnetic (*S* = 0) due to the preferred LS configuration of the Fe^2+^ ions. Pyrite’s natural abundance, optical band gap of ∼0.95 eV, and large optical absorption coefficient make it an appealing material for photovoltaic applications. Nevertheless, despite theoretical predictions suggesting an open circuit voltage of ∼0.71 V based on the Shockley-Queisser equations, experimental results have consistently fallen short, typically measuring values around 0.2 V.^130^ This discrepancy is among several reasons motivating an accurate quantum-mechanical description of pyrite’s ground state.

In early DFT studies, the LDA functional demonstrated exceptional accuracy in predicting the equilibrium volume, band gap, and relative energy levels of the S-3*p* bands compared to the VBM.^129,131^ PBE^120^ and AM05^132^ provide a qualitatively similar picture but underestimate the band gap by about 0.5 and 0.75 eV, respectively.^130^

**Figure 2:**
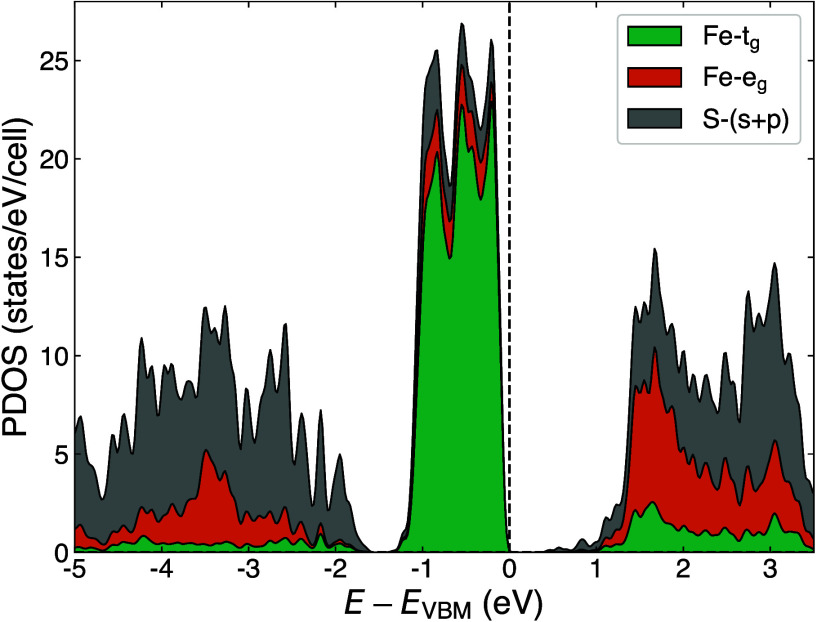
Stacked projected density of states (PDOS) of the nonmagnetic PBE ground-state of pyrite FeS_2_. The small *t*_*g*_ contributions^5^ in the conduction band and the *e*_*g*_ contributions close to the VBM are likely projection artifacts resulting from the slightly canted geometry of the FeS_6_ octahedra.

**Figure 3:**
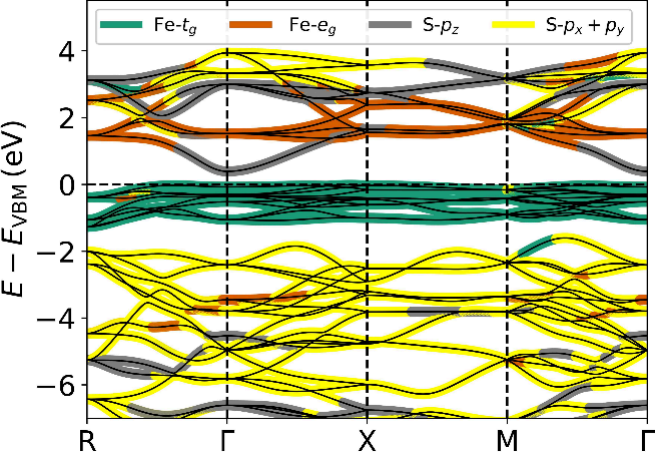
(Fat-)Band structure of pyrite computed using the PBE functional. The coloring indicates the most dominant contribution of the manifolds to the individual bands. The uppermost valence bands are predominantly Fe-*t*_*g*_, whereas the conduction bands display a strong S-3*p*_*z*_ character at Γ but Fe-*e*_*g*_ character elsewhere. Thus, the band gap of pyrite is not a *d*-*d* transition, but a *d*-*p* one.

At first glance, the dominant *e*_*g*_ contributions in the conduction band (Figure 2) indicate Mott-Hubbard insulation. However, a more detailed analysis of the electronic band structure (Figure 3) reveals that the CBM, located at the Γ point, is composed of a *d*–*p*σ* hybrid orbital with dominant S-3*p*_*z*_ contributions.

This observation holds the key to understanding why numerous electronic structure methods, including more advanced approaches, face challenges in improving the prediction of the band gap beyond the capabilities of PBE. For instance, when employing *G*_0_*W*_0_, the small PBE band gap diminishes further.^134^ In contrast, hybrid functionals such as PBE0,^135^ HSE06,^136^ DSH,^137^ and M06^138^ overestimate its value by more than 1 eV.^139^ Previous PBE+*U* studies focused on correcting the Fe-*d* shell using shell-averaged Hubbard parameters. For example, Sun et al. applied *U* = 2.0 eV (with PAW projectors),^130^ whereas Schena et al. used a combination of *U* = 3.0 eV with *J* = 1.0 eV (with Muffin-Tin sphere projectors).^134^ Notably, no set of *ab initio* computed Hubbard parameters has been published for this material to date, to the best of our knowledge. The rationale behind the use of empirical parameters likely lies in a pronounced influence of shell-averaged *U* corrections on the equilibrium properties, as we demonstrate hereinafter.

#### 4.1.2 Double Impact of Shell-Averaged *U* Corrections

We perform PBE+*U* calculations incorporating empirical on-site Hubbard *U* corrections ranging from 1.0 to 5.0 eV and analyze their impact on the estimated band gap and on the crystal structure. To differentiate between a band gap broadening caused by structural changes and one resulting directly from the influence of *U* on the electronic structure, we perform the calculations under three differently constrained conditions. Initially, we retain the PBE ionic structure, keeping the cell vectors and ionic positions fixed (setup 1). Subsequently, we perform another set of calculations with ionic position relaxations while maintaining a constant cell volume (setup 2). Lastly, we conduct full optimization, allowing for simultaneous adjustments of both ionic positions and cell vectors (setup 3). While band gaps are fundamentally outside the realm of DFT, DFT+*U* should improve upon the performance of the uncorrected functional as long as the compound’s frontier states are well-represented by the Hubbard manifold.^50,63^ For this case, one can argue that the Hubbard *U* corrections (locally) act in the spirit of a Koopmans-compliant functional.^17^

**Figure 4:**
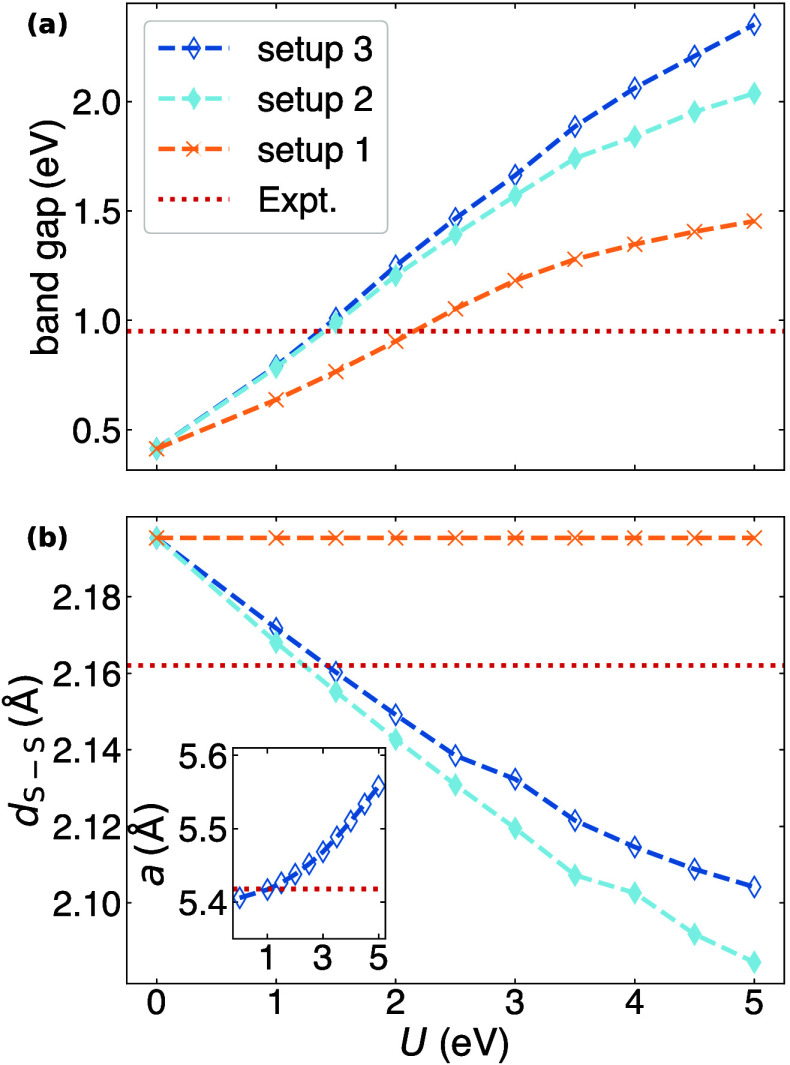
(a) calculated band gaps and (b) S–S bond lengths computed with PBE+*U* using three setups: at fixed cell and ionic positions (setup 1); at constant cell volume relaxing the ionic positions (setup 2); and relaxing both the ionic positions and the cell volume (setup 3). The inset in the bottom panel shows the dependence of the lattice parameter on *U* during volume relaxations. Red dashed horizontal lines correspond to the experimental values.^128,140^

Figure 4 illustrates that even when no ionic relaxation is considered (setup 1), the band gap slowly but steadily expands with increasing *U* values. The shifts in band eigenvalues indicate that this expansion primarily stems from a downshift of the *t*_*g*_ orbitals’ KS potential in the valence region (see Table SI 2). On the other hand, the CBM remains largely unaffected by the Hubbard correction with the corresponding eigenvalues showing minimal changes. Consistent with previous studies, we find that the experimental band gap is accurately reproduced at *U* ≈ 2.0 eV, but is overestimated by approximately 53% at *U* = 5.0 eV. This overestimation of the band gap at such moderate values of *U* is surprising since the CBM, consisting of S-3*p*_*z*_ states, is not included in the Hubbard manifold, and its local deviation from PWL remains uncorrected. Upon relaxing the ionic positions while keeping the cell volume constant (setup 2), the band gap shows a more sensitive response to higher values of *U*. The experimental band gap is already achieved at *U* ≈ 1.5 eV and is overestimated by 115% at *U* = 5.0 eV. This trend becomes even more pronounced when relaxation of the cell vectors is enabled (setup 3), resulting in a band gap overestimation of 147% at *U* = 5.0 eV. As shown in Figure 4(b), the additional expansion following ionic relaxations is correlated with a significant contraction of the S– S bonds. This aligns well with the findings of Eyert et al., who pointed out that the S–S bond length governs the band gap, as it controls the dispersion of the lowest conduction band around the Γ point.^131^ Remarkably, in variable-cell calculations (setup 3) the S–S bonds continue to contract with increasing values of *U*, despite the simultaneous growth of the lattice parameter (inset of Figure 4b).

The differences observed between relaxed and fixed structure calculations reveal that shell-averaged *U* corrections modify the band gap through two distinct mechanisms: first, through the expected downshift of the valence band eigenvalues, and second by causing pronounced contractions of the S–S bonds.

We now shift our focus from conventional equilibrium properties to investigate the underlying reasons behind the substantial influence of shell-averaged *U* corrections on these bonds, which can be elucidated by examining the occupations of the individual orbitals. Table 1 presents the eigenvalues of the Fe-*d* occupation matrix and the corresponding Hubbard energies *E*_*U*_ for an illustrative case with *U* = 3.0 eV, both prior to and after complete structural relaxation. As the eigenvalues of the *t*_*g*_ eigenstates (*v*_3_, *v*_4_, *v*_5_) approach idempotency, this manifold is responsible for the smaller share of the overall Hubbard energy, approximately 33%. In contrast, the *e*_*g*_ eigenstates (*v*_1_ and *v*_2_) contribute to nearly 66% of the total Hubbard energy due to possessing occupation eigenvalues far from 0 or 1. We note in passing that the *t*_*g*_ eigenstates strongly resemble the (hydrogenic) Löwdin atomic orbitals *d*_*xy*_, *d*_*xz*_ and *d*_*yz*_, whereas *v*_1_ and *v*_2_ are less well-represented by the atomic orbitals  and *d*_*z*^2^_ because of significant hybridization with neighboring S ligand orbitals. Structural relaxation induces both intrashell and intershell charge transfers that allow for a 6% reduction of the total Hubbard energy. At the intrashell level, the occupation of *t*_*g*_ orbitals grows at the expense of *e*_*g*_. Even more significantly, the overall *d*-occupancy slightly drops from 6.996 to 6.970, indicating a migration of some *e*_*g*_ electrons into adjacent S-*p* orbitals. This transfer of charge effectively enhances the S– S bond order and leads to the observed contraction of *d*_S–S_. Thus, the unexpectedly potent influence of the value of *U* on the band gap is rooted in the correction of the *e*_*g*_ manifold.

**Table 1:** Occupation Eigenvalues λ to Eigenstates *v* of the Fe-*d* Occupation Matrix and Corresponding Hubbard Energies *E*_*U*_ for the PBE+*U* Ground State of Pyrite with *U* = 3.0 eV^a^

Representation		*e*_*g*_		*t*_*g*_			2Σ
eigenstate		*v*_1_	*v*_2_	*v*_3_	*v*_4_	*v*_5_	
setup 1	λ	0.383	0.383	0.901	0.901	0.930	6.996
	*E*_*U*_ (meV)	354	354	134	134	98	2148
setup 3	λ	0.359	0.359	0.915	0.915	0.937	6.970
	*E*_*U*_ (meV)	343	343	117	117	89	2018

a Results are shown for the fixed PBE structure (setup 1, *a* = 5.406 Å and a S– S distance of 2.195 Å, top) and after structural relaxation (setup 3, *a* = 5.469 Å and a S– S distance of 2.132 Å, bottom). The last column shows the total occupations (2*∑λ*) and Hubbard energies (2*∑E*_*U*_), which correspond to twice the sum (due to spin degeneracy) of the individual contributions.

We recall that the eigenvalues listed in Table 1 are not universal quantities but the result of a projection of the KS wave functions onto atom-centered OAO. The accuracy of on-site occupations provided by atomic-like projectors relies on the similarity between a given orbital’s shape in the system under inspection and that in a free atom, since the general shape and extension of projector AOs is typically determined in free atom calculations. However, one-center projections using atomic orbitals struggle to account for strong orbital hybridization, where electrons localize “off-site” between the bonded atoms. In the case of FeS_2_, the *t*_*g*_ manifold is only marginally bonding, while the formally unoccupied *e*_*g*_ orbitals form σ MOs with neighboring S-3*p*_*z*_ orbitals. Consequently, it is misleading to interpret the eigenvalues determined for the *e*_*g*_ states (*v*_1_ and *v*_2_) as indicative of actual on-site orbital occupancies in the context of Hubbard *U* corrections. Moreover, their numerical values significantly hinge on computational factors like the chosen pseudopotentials and the specific charge state for which they were parametrized.^62^

Within the setup of this study, the *U* correction effectively penalizes the hybridization between Fe and S; however, the use of different Hubbard projector functions might cause the the opposite effect, for example, if NAO projectors yield *e*_*g*_ eigenvalues larger than 0.5. In this case, the Hubbard correction would draw electrons into the *e*_*g*_ manifold rather than expelling them (see Eq. 3).

#### 4.1.3 Absence of Intrashell Screening in Shell-Averaged LR-cDFT

Having investigated how the shell-averaged *U* parameter affects equilibrium observables, our focus now shifts to understanding the implications when this parameter is determined from first principles. Applying DFPT to the PBE ground state of FeS_2_ yields a *U* value of 7.37 eV. After undergoing four iterations within the self-consistency loop detailed in Section 2.3, *U* converges to 6.47 eV. Unfortunately, neither of these parameters can reasonably reproduce the experimental characteristics of pyrite. In fact, both values result in the stabilization of a spurious ferromagnetic ground state (2 μ_*B*_/cell) in unrestricted open-shell calculations. For comparison, all of the following *U* parameters and observables are reported for the nonmagnetic ground state that was enforced by fixing the total magnetization to 0.0 μ_B_.

**Figure 5:**
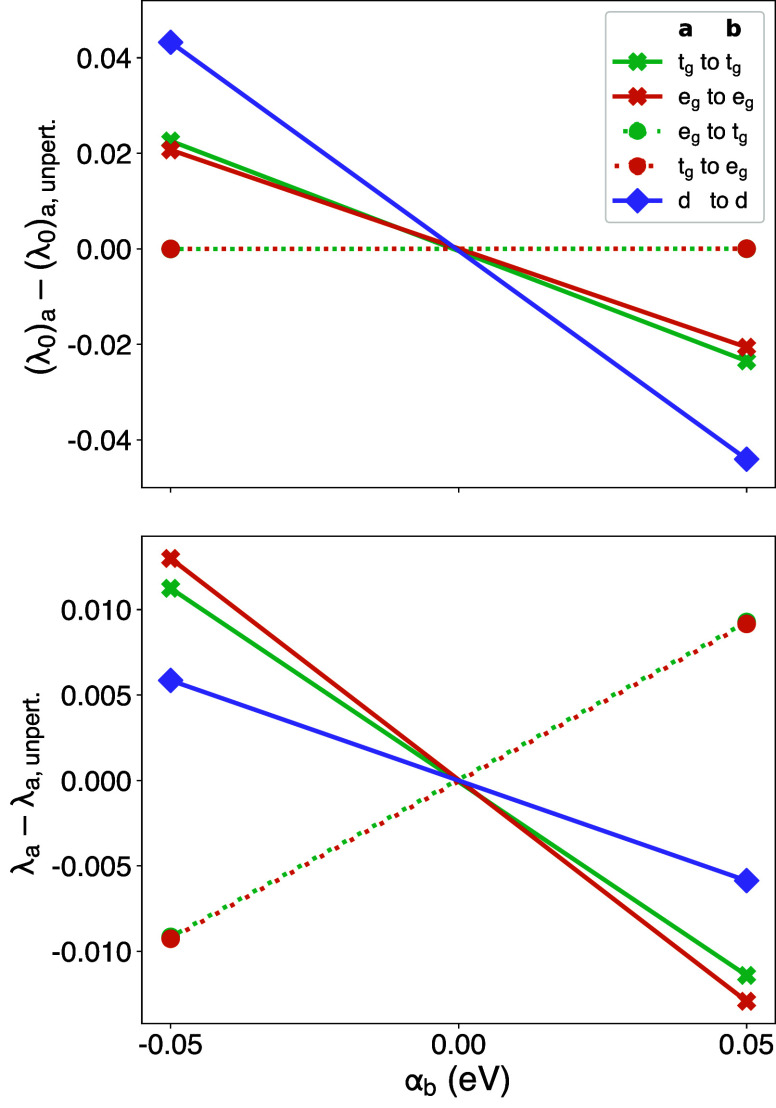
Unscreened/noninteracting (top) and screened/interacting (bottom) responses of the occupations λ of manifolds “**a**” to perturbations α applied to manifolds “**b**” in FeS_2_, obtained through the orbital-resolved LR-cDFT approach. The starting point is the fully relaxed PBE ground state.

The reason for this significant overestimation of the shell-averaged Hubbard *U* parameter can be understood from Figure 5, which shows the response of orbital occupancies to perturbations for both the entire 3*d*-shell and for its irreducible representations *t*_*g*_ and *e*_*g*_. This information cannot be extracted from shell-averaged LR-cDFT, but can be recovered by adopting the orbital-resolved approach. The substantial opposing responses of *e*_*g*_ occupations to perturbations of *t*_*g*_ and vice versa (represented by dotted lines) suggest the presence of a robust intrashell *t*_*g*_ ↔ *e*_*g*_ screening channel. Inserting the values of χ_0_ and χ (represented by the line slopes in Figure 5) into eq 16, one obtains
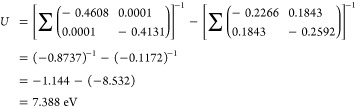
17

where the first and second matrix represent the unscreened and the screened responses, respectively. Note the almost perfect agreement between this LR-cDFT Hubbard parameter (*U* = 7.39 eV) and the one derived from DFPT (*U* = 7.37 eV); the minor discrepancies are due to numerical noise. The individual matrix elements of eq 17 reveal that the apparent overestimation of the shell-averaged Hubbard parameter primarily stems from the suppression of intrashell screening that occurs due to the simultaneous perturbation of the *t*_*g*_ and the *e*_*g*_ manifolds. While the individual screened responses of the *t*_*g*_ and *e*_*g*_ orbitals are substantial ( and ), the overall response of the *d* shell is massively reduced by the off-diagonal elements of χ (both 0.1843 eV^–1^). Inversion of this *apparently* small screened response of the *d* shell (−0.1172 eV^–1^) yields χ^–1^ = −8.532 eV, which is what ultimately fuels the overestimation of the shell-averaged Hubbard parameter.

It should be noted that this shell-averaged *U* parameter also encompasses, to a certain extent, the response of the S-3*p* orbitals, because the *e*_*g*_ eigenstates likely contain substantial S-3*p* contributions. With these insights in mind, it becomes pertinent to investigate whether the orbital-resolved approach to Hubbard *U* can offer improvements over the unsatisfactory performance of shell-averaged PBE+*U*.

#### 4.1.4 Application of the Orbital-Resolved *U*

Employing the orbital-resolved LR-cDFT methodology detailed in Section 2.3, we proceed to compute individual Hubbard parameters for the *t*_*g*_ and *e*_*g*_ orbitals of FeS_2_. Prior to inverting the response matrix, all off-diagonal intrashell matrix elements are set to zero (χ_0_ is set to zero only to avoid numerical noise):
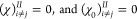
18

This ensures that the resulting orbital-resolved Hubbard parameters incorporate the effect of *t*_*g*_ ↔ *e*_*g*_ screening. The parameters *U*_*t*_*g*__ and *U*_*e*_*g*__ converge rapidly, reaching their self-consistent values of 3.29 and 2.16 eV, respectively, within just three iterations of self-consistency. The final response matrices read
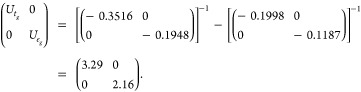
19

Following the approach of Solovyev et al., one may also exclusively target the *t*_*g*_ manifold and not correct the *e*_*g*_ states at all. This choice is driven by the expectation that interactions of genuine *on-site* character should manifest within the occupied *t*_*g*_ orbitals rather than in the hybridized and formally empty *e*_*g*_ manifold. Moreover, as previously stated, it is unlikely that one-center atomic orbital projectors are suited to provide meaningful on-site occupation numbers for the *e*_*g*_ orbitals. With *U*_*e*_*g*__ corrections absent, the converged value of *U*_*t*_*g*__ slightly decreases to 3.01 eV.

Having established the necessary parameters, we now focus on their impact in practical calculations. Table 2 shows that the orbital-resolved approach consistently outperforms the shell-averaged approach, except when *U* is tuned empirically (*U*^emp^). When utilizing only *U*_*t*_*g*__, both the lattice parameter and the S– S bond length exhibit deviations of less than 1% from the experimental values. With orbital-resolved corrections applied to *t*_*g*_ and *e*_*g*_, this agreement with the experimental data is slightly worsened but still surpasses that of shell-averaged PBE+*U*, where the lattice parameter deviates by 4%. Furthermore, the significant overestimation of the band gap amounting to 177% in the case of shell-averaged PBE+*U* is substantially mitigated to 53% when switching to *U*_*t*_*g*__. The DFT+*U*+*V* approach yields noticeable improvements compared to the traditional + *U* approach; however, it appears that the intersite *V* term lacks the strength to fully restore the hybridization between Fe and S that is suppressed by the shell-averaged on-site term. Therefore, the estimation of the lattice parameters and the band gap still remains notably worse than for the orbital-resolved PBE+*U* approach.

**Table 2:** Comparison of the Equilibrium Lattice Parameter *a*, the S–S Bond Length *d*_S–S_, and the Band Gap *E*_g_ of Pyrite Derived from Hubbard-Corrected PBE Calculations Targeting Different Manifolds^a^

	PBE	+*U*	+*U*+*V*	+*U*^emp^	+*U*_*t*_*g*__+*U*_*e*_*g*__	+*U*_*t*_*g*__	Expt.
*U*_1_(eV)	—	6.47	6.73	1.50	3.29	3.01	—
*U*_2_ or *V*(eV)	—	—	0.62	—	2.16	—	—
*a*(Å)	5.41	5.63	5.51	5.43	5.46	5.44	5.42^128^
*d*_S–S_(Å)	2.20	2.10	2.12	2.16	2.13	2.14	2.16^128^
*E*_g_(eV)	0.41	2.63	2.06	1.00	1.70	1.45	0.95^140^

a On-site Hubbard *U*_1_ and *U*_2_ refer to various parametrizations of the PBE+*U* approach, while *V* is the inter-site Hubbard parameter of the PBE+*U*+*V* approach.

These results underscore the critical importance of the choice of the Hubbard manifold in DFT+*U* calculations for the FeS_2_ polymorph pyrite. Under the shell-averaged approximation, the ground-state properties are extremely sensitive to the numerical value of *U*. This sensitivity primarily arises due to the correction of the hybridized *e*_*g*_ part of the *d* shell. Moreover, the shell-averaged Hubbard parameter derived from LR-cDFT lacks full screening, as the intrashell screening channel (*t*_*g*_ ↔ *e*_*g*_) is deactivated during the concurrent perturbation of both manifolds. On the other hand, the orbital-resolved approach accounts for intrashell screening during perturbative calculations, resulting in significantly smaller Hubbard parameters. We have shown that correcting the localized Fe-*t*_*g*_ manifold alone suffices to obtain structural properties in very good agreement with experimental data, all without requiring empirical adjustments to the parameters. A viable option for further improvements involves supplementing the orbital-resolved *U* corrections with intersite *V* terms, as discussed in Section 5. Additionally, the PWL condition should also be enforced for the S-3*p*_*z*_ orbital composing the CBM. Given the strong *sp*^3^ hybridization of S, this would, however, require an adaption of the atomic-like projectors used in this study.

### 4.2 Fe(II) Molecular Complexes

#### 4.2.1 Adiabatic Spin Energy Differences

A crucial quantity for many TM compounds is the total energy difference between their HS and LS states, denoted as
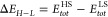
20

Based on calculations of six Fe(II)-hexacomplexes, Mariano et al. showed that shell-averaged Hubbard *U* corrections to PBE and LDA introduce an unphysical bias against LS configurations, primarily due to substantial discrepancies in the Hubbard energies of LS and HS complexes.^84^ This arises from the electron occupation patterns: LS-Fe(II) complexes exhibit a *t*_2*g*_^6^/*e*_*g*_^0^ electron configuration, while HS complexes a *t*_2*g*_^4^/*e*_*g*_^2^ configuration. In octahedral coordination environments, *unoccupied e*_*g*_ orbitals display substantial σ overlap with neighboring ligand orbitals. Consequently, in LS-Fe(II) all four *e*_*g*_ spin–orbitals can adopt fractional occupation eigenvalues, whereas in HS-Fe(II) this only applies to two spin–orbitals, as the other two are fully occupied. Considering that the Hubbard energy functional (eq 1) induces corrections of up to *U*/2 eV for fractional occupations, but of 0 eV for idempotent occupations, Hubbard energies of LS-Fe(II) tend to be larger than those of HS-Fe(II). Furthermore, the LR-cDFT approach was shown to generate larger shell-averaged *U* parameters for LS-Fe(II) compared to HS-Fe(II),^84^ thus enhancing this disparity.

In the following, we investigate whether the orbital-resolved DFT+*U* approach can rectify the unphysical bias against LS states and deliver accurate adiabatic spin energy differences. For this purpose, we compute Δ*E*_*H*–*L*_ for all six octahedrally coordinated Fe(II)-hexacomplexes studied in ref (84) and benchmark the results against coupled-cluster corrected CASPT2 (CASPT2/CC)^141^ values, which are also taken from ref (84). We compare the performance of two distinct Hubbard manifolds. In **manifold I**, Hubbard corrections are applied either only to *e*_*g*_ states ( and ) or to only *t*_2*g*_ states (, , , ). While it would be desirable to obtain *t*_2*g*_-specific Hubbard parameters for the former two compounds, this proves elusive due to the fact that the *t*_2*g*_ orbitals are fully occupied in the numerical sense (λ ≥ 0.997) and thus respond nonlinearly to perturbations.^113^ For the same reason, no  parameters could be computed for the HS complexes either. Therefore, all Hubbard corrections to *t*_2*g*_ states utilize the  value. In **manifold II**, both *t*_2*g*_ and *e*_*g*_ orbitals are corrected simultaneously. When comparing the present work with ref (84), it is important to recall our use of OAO projector functions and geometry optimizations, whereas in ref (84) NAO projector functions were used and the geometries were not optimized. A comprehensive list of all complexes, the Hubbard manifolds considered and the corresponding orbital-resolved and shell-averaged *U* parameters is provided in Table 3.

**Table 3:** PBE+*U* Manifolds and Their Calculated On-Site Parameters in eV^a^

Complex	manifold I	manifold II	*U*_t2g_^LS^	*U*_eg_^LS^	*U*_eg_^HS^	*U*^LS^	*U*^HS^
[Fe(H2O)_6_]^2+^	*e*_*g*_	—	—	4.28	2.69	5.71	4.13
[Fe(NH3)_6_]^2+^	*e*_*g*_	—	—	3.64	3.07	6.14	4.43
[Fe(NCH)_6_]^2+^	*t*_2*g*_	*t*_2*g*_+*e*_*g*_	7.05	2.12	1.87	6.76	5.53
[Fe(PH3)_6_]^2+^	*t*_2*g*_	*t*_2*g*_+*e*_*g*_	4.13	2.29	2.12	6.88	4.78
[Fe(CO)_6_]^2+^	*t*_2*g*_	*t*_2*g*_+*e*_*g*_	4.63	1.92	1.89	7.16	5.43
[Fe(CNH)_6_]^2+^	*t*_2*g*_	*t*_2*g*_+*e*_*g*_	4.64	2.13	1.72	7.43	5.79

a For *t*_2*g*_ the same parameter was applied to LS and HS complexes. The complexes are sorted in ascending order according to the ligand’s field strength.

**Figure 6:**
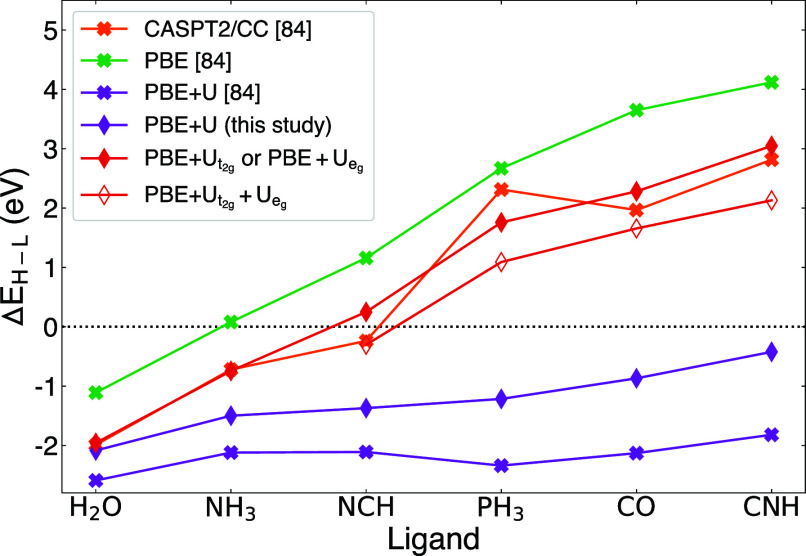
Adiabatic spin energies calculated for six Fe(II)-hexacomplexes using different DFT corrections and CASPT2/CC as the reference method. Data for PBE, PBE+*U*, and CASPT2/CC was taken from the SI of ref (84).

The resulting adiabatic spin energy differences are visualized in Figure 6. Additionally, Table 4 lists the mean absolute error (MAE) of the different corrections against the CASPT2/CC^141^ reference values. In line with the findings of ref (84), shell-averaged PBE+*U* drastically overstabilizes HS configurations and fails to reproduce the trend of steeply increasing Δ*E*_*H*–*L*_. Conversely, uncorrected PBE displays its known bias toward LS configurations. The introduction of orbital-resolved *U* corrections substantially enhances the predictive accuracy across all complexes analyzed, outperforming both PBE and the shell-averaged PBE+*U* by a large margin. Correcting either *t*_2*g*_ or *e*_*g*_ (manifold I) closely aligns adiabatic spin energy differences with the CASPT2/CC values, resulting in the lowest MAE of 0.27 eV. The simultaneous correction of *t*_2*g*_ and *e*_*g*_ (manifold II) yields a slightly higher MAE of 0.57 eV, with larger deviations particularly for  and , but a better agreement for . In general, *U*_*e*_*g*__ corrections are smaller and less significant for the overall quantitative agreement than *U*_*t*_2*g*__ corrections, since the *t*_2*g*_ orbitals host more–and more localized–electrons. The good overall agreement (qualitative *and* quantitative) of orbital-resolved PBE+*U* with the CASPT2/CC data is surprising since the latter is a multiconfigurational approach, while the former can only improve the accuracy of a single reference state.

**Table 4:** Mean Absolute Error (MAE) of Δ*E*_*H*–*L*_ for Various Hubbard Correction Manifolds against the CASPT2/CC Reference Value from Ref (84)

	PBE^84^	+*U*	+ *U*_*t*_2*g*__ or + *U*_*e*_*g*__	+ *U*_*t*_2*g*__ + *U*_*e*_*g*__
MAE (eV)	1.07	1.94	0.27	0.57

The good performance of the orbital-resolved *U* compared to the shell-averaged approach stems from two main factors. First, excluding the *e*_*g*_ orbitals from the Hubbard manifold of strong-field complexes (manifold I) eliminates the primary cause of spuriously large Hubbard energies. Again, the exclusion of *e*_*g*_ orbitals can be justified with the inadequate representation of their strongly hybridized character by atomic orbital projectors. Second, even when the *e*_*g*_ orbitals are included (manifold II), independently computed *U*_*e*_*g*__ parameters are significantly smaller than *U*_*t*_2*g*__ or shell-averaged *U* parameters owing to the explicit incorporation of intrashell screening. For instance, in  eV compared to *U*^LS^ = 7.16 eV. Hence, the orbital-resolved approach effectively mitigates the impact of nonideal Hubbard projectors.

#### 4.2.2 Piecewise Linearity of the Total Energy

As mentioned earlier, the use of on-site Hubbard *U* corrections in (semi)local DFT is motivated by the mitigation of SIE, which have been linked to the spurious global deviation from PWL of the total energy with respect to fractional addition or removal of electronic charge *q* to the entire system.^12,15,18^ However, a study by Zhao et al. indicates that (shell-averaged) *U* corrections to TM *d* shells might be unfit for this purpose for a wide range of TM/ligand combinations when the Hubbard *U* parameters are derived from LR-cDFT.^15^

Thus, to directly benchmark the performance of orbital-resolved DFT+*U*, we explicitly determine the global curvature following the approach presented in ref (15). For our analysis, we chose the strongest ligand complex [Fe(CNH)_6_]^q+^ and perform several fixed-charge calculations where the total charge is varied between *q* = 2 and *q* = 3 in increments of 0.1e^–^.

A useful metric to assess a functional’s deviation from PWL is given by *E*_dev_, which is computed by subtracting the DFT total energy of a fractional charge calculation from the linear interpolation between the energies of the integer-charge end points *E*_*tot*_(*q* = 2) and *E*_*tot*_(*q* = 3). Note that this explicit approach differs from the method employed in ref (84), where *E*_dev_ is approximated using a cubic interpolation parametrized by the energy difference Δ*E*_*q*_ = *E*_*tot*_(*q* = 2) – *E*_*tot*_(*q* = 3) and the HOMO and LUMO eigenvalues of the integer-charge system.

**Figure 7:**
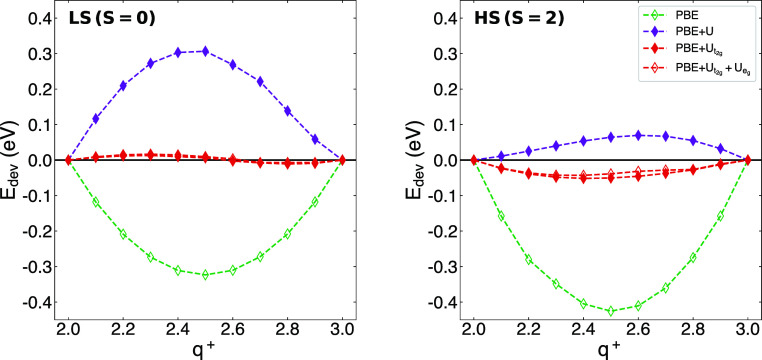
Deviation from piecewise linearity of the total energy upon fractional addition/removal of charge of [Fe(CNH)_6_]^q+^ in low-spin (left) and high-spin (right) configurations. Note that the data points of PBE + *U*_*t*_2*g*__ and PBE + *U*_*t*_2*g*__+*U*_*e*_*g*__ overlap in the LS case.

Figure 7 shows that while shell-averaged Hubbard *U* corrections markedly reduce the deviation from PWL of HS [Fe(CNH)_6_]^q+^, the global curvature of the LS compound is not eased. In fact, in the LS case the typical convex-shaped FCE displayed by bare PBE is transformed into a concave one of similar magnitude. In contrast, the orbital-resolved corrections reduce the deviation from PWL by an order of magnitude for both the LS and the HS configurations. It is worth noting that the remaining curvature barely differs between manifold I (+*U*_*t*_2*g*__ only) and manifold II (+*U*_*t*_2*g*__ + *U*_*e*_*g*__). Again, this observation is likely related to the low numerical value of the *U*_*e*_*g*__ parameters, which further supports the previous assumption that the primary contributor to the FCE is the rather localized *t*_2*g*_ manifold. For the same reason, applying *U* corrections to ligand orbitals like C-*p* is unlikely to significantly affect the FCE.

### 4.3 The Importance of Correcting Ligand States in β-MnO_2_

Unlike in TM sulfides, partially filled *d* shells of TM oxides are a frequent target for on-site corrections (see the discussion e.g. in ref (79)). While Mott-Hubbard insulators such as the prototypical wüstite (FeO) present an energy gap separating different *d* states, charge-transfer insulators, often involving highly oxidized and/or heavy TM species, exhibit O-2*p* → *d* band gaps.^142^ The significant fraction of electrons localized on O-2*p* orbitals adds complexity to the definition of the Hubbard manifold for DFT+*U* calculations, as studied below for the case of β – MnO_2_.

Crystallizing in the rutile structure (space group *P*4_2_/*mnm*), β – MnO_2_ displays a complex helical (screw-type spiral) antiferromagnetic (AFM) ordering below *T*_*N*_ = 92K.^143^ As done here, this ordering can be approximated by a collinear arrangement of the Mn^4+^ ions along [001], referred to as A1-AFM.^59^ Considerable uncertainty surrounds the electronic properties of β – MnO_2_. Sato et al. measured a large electrical resistivity at 0.3 K, suggesting insulating behavior.^143^ However, the magnitude of the band gap has not been yet determined with universally accepted accuracy. Midtwentieth-century works reported narrow band gaps around 0.26–0.28 eV,^144,145^ while more recent optical absorption studies reported much larger values ranging from 1.5 eV (nanocacti and nanorods)^146^ to 2.0 eV for β – MnO_2_ nanostructures grown on fluorine-doped tin oxide.^147^ These results align with a reported hybrid functional DFT (PBE0) value of 1.5 eV, but not with a prediction of the closely related HSE03 functional (0.6 eV).^148^ Previous works also indicate that the opening of a band gap is relatively insensitive to shell-averaged Hubbard *U* corrections to the Mn-*d* states.^59,148^ For example, in ref (59) the use of a self-consistent *U* parameter of 6.34 eV only induced an extremely small gap of 0.02 eV in the A1-AFM case (using orthogonalized atomic Hubbard projectors). In general, the calculated properties are sensitive to the choice of projector functions. For instance, NAO projectors stabilize a ferromagnetic ordering, whereas OAO projectors favor the expected A1-AFM ordering.^59^ Only by adding intersite *V* between Mn-*d* and O-*p* states^59^ or Hund’s *J* corrections,^149^ small gaps ranging from 0.25 to 0.32 eV emerge. A band gap of 0.8 eV was also obtained^91^ with the anisotropic DFT+*U* + *J* approach of Czyżyk and Sawatzky. In this method, shell-averaged Hubbard *U* and *J* corrections are augmented with orbital-resolved *U*_*mm*′_ and *J*_*mm*′_ matrices parametrized from summation relations involving Slater integrals of atomic Hartree–Fock calculations.^88^ This approach thus differs from the here-presented scheme, which does not rely on model systems like the free atom and includes intrashell screening effects, such as *t*_2*g*_ ↔ *e*_*g*_.

The wide range of band gap values reported in both the theoretical and experimental literature underscores the pressing need for a more profound understanding of the insulating behavior of β – MnO_2_. Considering the charge-transfer nature of the band gap and the coexistence of relatively localized and strongly hybridized states in both the Mn-*d* shell and the O-*p* shell, orbital-resolved DFT+*U* calculations offer a promising starting point. In the interest of comparability, our calculations are carried out using a setup equivalent to that of ref (59), imposing the A1-AFM ordering in a 2 × 2 × 2 supercell containing 48 atoms and considering once again different Hubbard manifolds.

**Figure 8:**
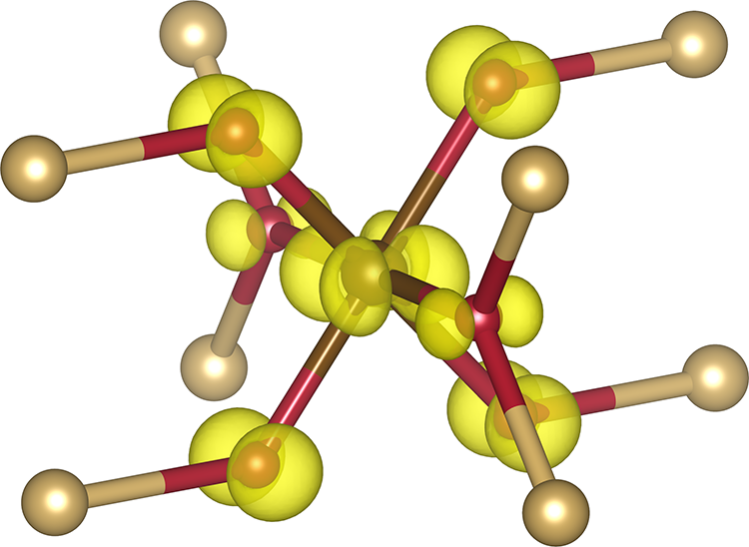
Isosurface plot of the highest fully occupied KS band of A1-AFM β – MnO_2_ obtained using PBEsol , showing π interactions between O-*p*_*z*_ and Mn-*t*_2*g*_ orbitals. Mn atoms of opposite spin-polarization are colored dark and light brown, O atoms are red. For clarity, the interaction is only shown for one MnO_6_ octahedron.

The first manifold solely considers the *t*_2*g*_ orbitals in order to single out the impact of the localized Mn-*d* states.^6^ As shown in Table 5, this correction only marginally influences the band gap or the structural properties in comparison to bare PBEsol. This observation aligns with the modest value of  eV, indicative of minimal local curvature due to the *t*_2*g*_ states. A much more significant improvement is achieved by considering the localized O-*p*_*z*_ orbitals (Figure 8). Correcting these states with a self-consistent Hubbard parameter  eV (in addition to the  correction) leads to an insulating gap of 1.01 eV. Moreover, the lattice parameters approach their experimental values, resulting in an error on the predicted cell volume smaller than 1%. Conversely, a shell-averaged *U* correction as large as 6.34 eV for Mn-*d* states only results in less accurate cell parameters and a still negligible band gap. This situation is only partly mended by the application of an additional intersite *V* parameter.^59^

**Table 5:** Comparison of Equilibrium Properties of A1-AFM β-MnO_2_ Obtained from PBEsol without and with Various Hubbard Corrections^a^

	PBEsol	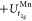	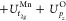	+*U*^Mn^ (Ref ^59^)	+*U*^Mn^+*V*^Mn–O^ (Ref ^59^)	Expt.
*U*_1_ (eV)	—	1.59	1.64	6.34	6.76	—
*U*_2_ or *V* (eV)	—	—	4.62	—	(0.99, 1.10)	—
*a* (Å)	4.37	4.37	4.38	4.40	4.39	4.40^150^
*c* (Å)	2.83	2.86	2.88	2.94	2.92	2.88^150^
*V*_0_ (Å^3^)	54.68	54.64	55.26	57.07	56.35	55.79^150^
*E*_g_ (eV)	0.00	0.00	1.01	0.02	0.32	0.26,^144^ 1.50,^146^ 2.0^147^

a *U*_1_ and *U*_2_ refer to various parameterizations of the PBEsol+*U* approach, while *V* is the inter-site Hubbard parameter of the PBEsol+*U*+*V* approach. *a* and *c* are the lattice parameters, *V*_0_ is the unit cell volume, and *E*_*g*_ is the band gap value. All the presented results (including those from ref (59)) were obtained using OAO projectors.

**Figure 9:**
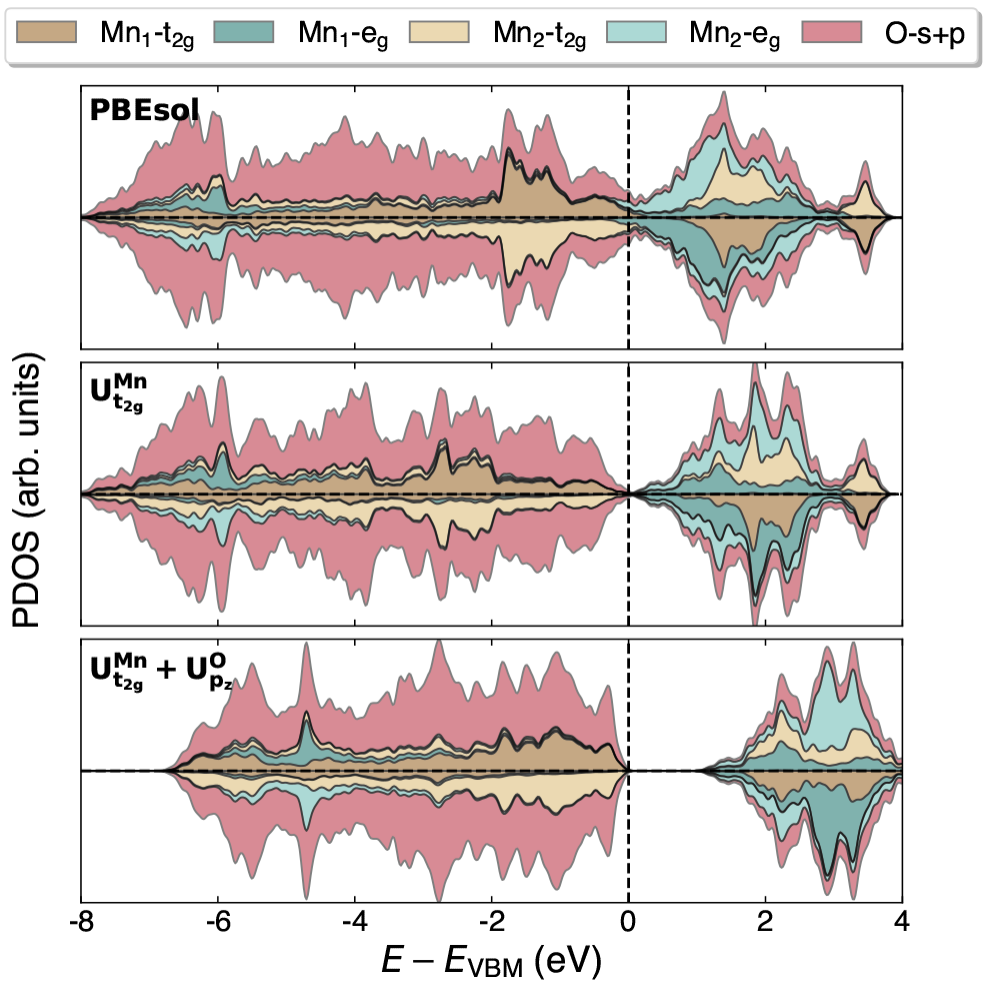
Stacked spin-resolved PDOS of the A1-AFM collinear ordering of β – MnO_2_ computed with PBEsol and two types of orbital-resolved *U* corrections. Metallic behavior is observed when using PBEsol and PBEsol , whereas an insulating character is obtained with PBEsol . On each panel, the upper and lower subpannels correspond to the spin-up and and spin-down channels, respectively. Mn_1_ and Mn_2_ represent symmetry equivalent atoms of opposite spin polarization (due to AFM ordering).

The importance of including O-*p*_*z*_ in the Hubbard manifold is evidenced by the PDOS depicted in Figure 9. The states near the Fermi level exhibit notable O-*p* contributions, regardless of the correction applied. These contributions primarily stem from localized *p*_*z*_ orbitals, which engage in π interactions with the Mn-*t*_2*g*_ manifold (Figure 8). The other two *p* orbitals are subject to *sp*^2^ hybridization and contribute to the formation of the lower-lying σ MOs in conjunction with Mn-*e*_*g*_ states. This observation suggests that applying shell-averaged *U* corrections to the entire O-*p* shell would weaken the σ bonds and lead to Mn–O underbinding. For the same reason, applying corrections to the Mn-*e*_*g*_ states is neither required nor useful.

In conclusion, the orbital-resolved approach pinpoints deviations from PWL in the localized O-*p*_*z*_ orbitals as the potential root-cause of the absence of a band gap in β – MnO_2_ when using DFT with semilocal functionals. This outcome underscores the frequently overlooked significance of ligand orbitals in electronic localization and establishes a foundation for future investigations. Again, achieving more comprehensive descriptions of this intricate charge-transfer insulator could entail incorporating the orbital-resolved *U* approach alongside intersite *V* and unlike-spin Hund’s *J* terms.

## 5 Outlook: Orbital-Resolved Intersite Hubbard Interactions?

Hubbard *U* corrections are primarily designed to mitigate the FCE in KS states exhibiting strong atomic-like character, that is, at the on-site level. Orbitals involved in strong interatomic hybridization should not be corrected using *U* terms. This limitation is a consequence of the one-center projectors that are typically used to determine the occupation numbers for the Hubbard *U* energy functional, which inherently lack the ability to adequately represent localization on hybrid (i.e., molecular) orbitals. To counteract the FCE within covalent environments, the extended DFT+*U*+*V* framework was introduced in ref (58), where conventional shell-averaged *U* corrections are augmented with an intersite term scaled by a Hubbard parameter *V* in a way that restores ligand hybridization through two-center (dual) occupation numbers. However, although the + *V* correction often improves the predictions of electronic and structural properties compared to plain DFT+*U*, the results presented for FeS_2_ and β-MnO_2_ exemplify that it only partially mitigates the impact of shell-averaged *U* corrections. Specifically, intersite terms cannot rectify a substantial bias in the total energy arising from on-site terms since the values of *U* (for first-row TM elements) are typically between 4 and 10 eV, which is several times larger than typical values of *V*, which amount to ≈1 eV. For instance, the Hubbard *U* and *V* parameters of the strong-field molecular complex  from Sec. 4 are 8.42 (for Fe-3*d* states) and 1.12 eV (between Fe-3*d* and C-2*p* states) for the LS configuration, and 6.57 and 1.11 eV for the HS configuration, respectively. Applying these parameters, one obtains an adiabatic spin energy difference Δ*E*_*H*–*L*_ = −0.42 eV that erroneously implies a HS ground state for  (the CASPT2/CC reference is +2.82 eV, see Figure 6).

Given that the majority of spurious contributions to the total energy arise from the shell-averaged form of *U*, the implementation of orbital-resolved DFT+*U*+*V* is an intriguing prospect, as already hinted in the concluding remarks of ref (58). This extension could exert precise control over electron localization on molecular orbitals, which could be achieved through individual Hubbard *V*_*ij*_^*IJ*^ parameters. Moreover, such highly tailored but still fully first-principles corrections should allow to eliminate potential conflicts arising from the simultaneous treatment of orbitals by both *U* and *V* terms. With regard to the compounds discussed in the present work, the orbital-resolved *U* corrections of the localized *t*_*g*_/*t*_2*g*_ orbitals could be augmented by *V* terms specific to the interaction of *e*_*g*_ with neighboring ligand orbitals. Note that orbital-resolved intersite *V* parameters are readily obtained as a byproduct of the calculation of *U* parameters within the generalized LR-cDFT approach.

However, practical calculations with this fully resolved approach would require the *a priori* assignment of two-center occupation numbers to specific MOs, causing additional preparatory effort. Desirable will be the conception and implementation of appropriate automation workflows in supporting tools such as AiiDA.^151,152^ Alternatively, a simplified approach could involve using the Hubbard *V* correction in its current (averaged) form while retaining the orbital-dependence of *U*. This would serve as a pragmatic *ad-hoc* solution to the issues of *U* at the expense of consistency.

Another avenue for extension pertains to Hund’s *J* corrections, whose combination with the orbital-resolved *U* should be straightforward. Such “unlike-spin” terms play a pivotal role in addressing the fractional spin error that occurs in systems characterized by significant magnetic coupling.^18,22,55,68,81,82,92^

## 6 Conclusions

In this study, we have introduced an orbital-resolved generalization of the DFT+*U* functional originally formulated by Dudarev et al. Our implementation is agnostic about the specific nature of the Hubbard projectors employed, and calculations involving forces and stresses require no adjustments stemming from this expansion. Despite not being a “rotationally invariant” approach in a strict sense,^7^ the method features invariance against basis vector rotations for practical purposes, since the corrections are applied to eigenstates of the occupation matrix (i.e., the correct linear combination of the orthogonalized atomic projectors). As long as these eigenstates are sorted based on their corresponding eigenvalues (which available solvers typically do), the present orbital-resolved DFT+*U* scheme will consistently target the same linear combination of projectors independently of the given orientation of the individual projectors. The true potential of the scheme emerges when the required Hubbard parameters are derived from first principles. For this purpose, we have employed an adapted version of the LR-cDFT approach, enabling perturbative calculations with orbital resolution. This approach intrinsically incorporates intra-shell screening, which often causes orbital-resolved *U* parameters to be significantly smaller than their shell-averaged counterparts. Provided a proper selection of the target manifold, the orbital resolution therefore enables a more surgical use of DFT+*U* that avoids overcorrections, as comparative calculations of six Fe(II) molecular hexacomplexes as well as of the charge-transfer insulators pyrite and pyrolusite demonstrate.

For instance, the orbital-resolved approach effectively addresses the bias in adiabatic spin energies toward HS states observed in shell-averaged DFT+*U*.^84^ Particularly noteworthy is its success in accurately predicting spin energies across a diverse spectrum of Fe(II) hexacomplexes, achieved through selective corrections of the highly localized *t*_2*g*_ orbitals. Explicit fractional charge calculations on Fe[CNH]_6_^2+^ suggest that these improvements are not coincidental: the orbital-resolved approach reliably counteracts the spurious global curvature of DFT with (semi)local functionals with respect to the fractional addition or removal of electrons, reducing the FCE by an order of magnitude. In contrast, shell-averaged Hubbard *U* corrections exhibit a mixed performance: while they effectively diminish the FCE in the HS states, their efficacy wanes when addressing the LS states. Here, instead of rectifying the FCE, the convex error characteristic of (semi)local DFT is converted into a comparably pronounced concave error. The results also suggest that a sole correction of the localized *t*_2*g*_ orbitals performs slightly better than a joint correction of *t*_2*g*_ and the rather hybridized *e*_*g*_ manifold, even when using orbital-resolved *U* parameters. The superiority of the orbital-resolved formulation over the shell-averaged approximation also extends to the charge-transfer insulators FeS_2_ and β – MnO_2_. The correct nonmagnetic ground state of the former is only stabilized when orbital-resolved Hubbard parameters are used. The magnitude of the large experimental band gap of the latter can only be achieved by applying a pinpointed Hubbard correction to the frontier O-*p*_*z*_ orbitals, whereas standard Hubbard *U* corrections to the *d* shell of Mn fail to open a significant band gap.

As these examples illustrate, the success of the orbital-resolved formulation predominantly originates from the exclusion of hybridized orbitals from the Hubbard manifold. From a theoretical perspective, the necessity to exclude hybridized orbitals is rooted in the very definition of *U* as an *on-site* term. However, practically implementing this definition poses challenges, particularly given that many DFT+*U* investigations rely on atomic-like orbitals as Hubbard projectors. This approach can lead to occupancy eigenvalues far from 0 or 1, especially in compounds featuring covalent bonds. These fractional values, however, are not related to the electron self-interaction, but arise from a one-center projector being applied to a two-center phenomenon, namely a molecular orbital. In such scenarios, the orbital-resolved approach provides an *ad-hoc* solution that allows to circumvent the potential conflict of on-site corrections with the intricacies of covalently bonded systems.

In a broader context, it is crucial to emphasize that the refined approach presented in this work constitutes just one among various potent (Hubbard) corrections available to DFT. As such, its effectiveness and ability to achieve consistent enhancements relies on a thoughtful and technically well-executed application. In practical terms, this is achieved by adopting a more nuanced strategy for the determination of Hubbard manifolds, tailored to the specific Hubbard projectors employed. Such a strategy should consider *localized* ligand orbitals for Hubbard corrections in situations where these act as frontier states. In addition, it might be desirable to address the FCE in hybrid (i.e., molecular) orbitals. For this purpose, the use of Wannier functions as Hubbard projectors or the adoption of an extended orbital-resolved DFT+*U*+*V* approach should be contemplated. A pivotal aspect of our future investigations will focus on developing a protocol to automate the choice of Hubbard manifolds based on measurable criteria. This step aims to streamline and standardize the approach’s application, ensuring its systematic and effective use across diverse systems and scenarios. The data used to produce the results of this work are available at the Materials Cloud Archive.^153^

## Supporting Information Available

A detailed description of the tracking routine and other technical details about the implementation of the orbital-resolved Hubbard *U* corrections, a demonstration of the influence of linear perturbations on the shape and extension of eigenstates of the occupation matrix and data on shifts of VBM and CBM KS eigenvalues in pyrite due to Hubbard corrections. (PDF)

## Footnotes

1 Note that this “double counting” must not be confused with the double counting term in DFT+*U* energy functionals derived from the Hubbard model.

2 Pickett et al. define the *d* shell-averaged Hubbard parameter (in *their* notation) as *U*_*dd*_=[∑_*a*,*b*=*t*_2*g*_,*eg*_(χ)_*a*,*b*_]^−1^, whereas in this work we use the definition of eq 16 that accounts for the noninteracting (bare) response.

3 This results from a physical necessity: if a perturbation to the potential of a manifold *a* causes the occupation of *a* to increase, the electrons must be borrowed from another manifold, whose orbital occupation will naturally decrease, since the global charge is kept constant in LR-cDFT.

4 We used Fe.pbe-spn-kjpaw_psl.0.2.1.UPF,^121^s_pbe_v1.4.uspp.F.UPF,^122^O.pbe-n-kjpaw_psl.0.1.UPF,^121^H.pbe-rrkjus_psl.1.0.0.UPF,^123^P.pbe-n-rrkjus_psl.1.0.0.UPF,^123^C.pbe-n-kjpaw_psl.1.0.0.UPF,^123^N.pbe-n-radius_5.UPF (THEOS pseudo) and mn_pbesol_v1.5.uspp.F.UPF(122) and O.pbesol-n-kjpaw_psl.0.1.UPF(121) for β – MnO_2_.

5 The point group symmetry of Fe in pyrite is *T*_*h*_ rather than *O*_*h*_; thus, the triply degenerate irreducible representation is correctly named *t*_*g*_ instead of *t*_2*g*_.^133^

6 Here, we use the *t*_2*g*_ irreducible representation in spite of the actual *D*_4*h*_ site symmetry of Mn^4+^ and therefore neglect the small energetic splitting between the *A*_1*g*_, *B*_1*g*_ and *B*_2*g*_ representations.

7 This is because the matrix that diagonalizes the occupation matrix does not necessarily also diagonalize the orbital-resolved *U* matrix due to the elimination of the off-diagonal elements in χ and χ_0_.
