# Supplementary material for: Orbital-Resolved DFT+U for Molecules and Solids
Source: J Chem Theory Comput. 2024 May 31;20(11):4824–43. doi: 10.1021/acs.jctc.3c01403 (PMC11171274; doi:10.1021/acs.jctc.3c01403)
Supplement: Supplementary file 1 — ct3c01403_si_001.pdf [file ct3c01403_si_001.pdf]

# Supporting information for: Orbital-resolved DFT+U for molecules and solids

Eric Macke,[*,†] Iurii Timrov,[‡,§] Nicola Marzari,[‡,¶] and Lucio Colombi Ciacchi[†]

†*Faculty of Production Engineering, Bremen Center for Computational Materials Science and MAPEX Center for Materials and Processes, Hybrid Materials Interfaces Group, University of Bremen, Am Fallturm 1, 28359 Bremen, Germany*

‡*Theory and Simulation of Materials (THEOS) and National Centre for Computational Design and Discovery of Novel Materials (MARVEL), École Polytechnique Fédérale de Lausanne, CH-1015 Lausanne, Switzerland*

¶*University of Bremen Excellence Chair, Bremen Center for Computational Materials Science*

§*Present address: Laboratory for Materials Simulations (LMS), Paul Scherrer Institut (PSI), CH-5232 Villigen PSI, Switzerland*

E-mail: emacke@uni-bremen.de

# Additional details on the practical application of orbital-resolved Hubbard $U$ corrections

Practical DFT calculations with orbital-resolved Hubbard $U$ corrections are implemented using eigenstates of the occupation matrix $n$. For example, the $d$ shell of an atom surrounded by an octahedral crystal field with $O_h$ point symmetry has three filled eigenstates ($\lambda \approx 1$) corresponding to $t_{2g}$ orbitals and two (at least partially) empty eigenstates ($\lambda \approx 0$), which represent the $e_g$ orbitals. Most eigenvalue solvers (including LAPACK's ZHEEV routine[1] used by Quantum ESPRESSO) output the eigenvalues in ascending order. Thus, the user can specify (in the input file) which eigenstates shall be targeted based on the eigenvalue indices. For the octahedral example case, one would assign a Hubbard parameter, say $U_{t_{2g}} = 3.0\,\text{eV}$, to the eigenstates 3,4 and 5 of the $d$ shell in order to target the $t_{2g}$ manifold. Then, in every SCF iteration, the code will compute the occupation matrix, diagonalize it, and finally compute the Hubbard energy $E_U$ as well as the Hubbard potential $\hat{V}_U$ using the equations presented in Sec. 2.1.

This procedure, however, requires information about the orbital ordering *before* the orbital-resolved DFT+$U$ calculation is started. Moreover, eigenstates might swap their indices throughout the course of the SCF cycle, e.g., as a result of the application of Hubbard corrections. In our implementation, the risk of eigenstates swapping their indices due to changes in the projected eigenvalues is handled by means of an "orbital-tracking" procedure (first described in Ref. 2) that uses the dot product between a set of reference eigenvectors and the eigenvectors of the current SCF iteration as a measure of similarity. Since the dot product of two identical normalized vectors is one while that of two orthogonal vectors is zero, this computationally inexpensive operation enables that a Hubbard parameter intended to be applied to a certain (reference) eigenstate will always tackle the current eigenstate that is most similar to this original reference eigenstate. In practice, the reference states can be obtained via two different procedures:

1. If the orbital-resolved DFT+$U$ calculation restarts from a (pre-)converged charge density (e.g., the PBE one), the eigenvectors of this converged ground state are directly used as references. This is the stable and safe choice, because it allows the user to analyze the orbital ordering and thoroughly define the Hubbard manifold *before* applying the orbital-resolved $U$ corrections. This "two-step" procedure can be crucial to converge "tricky" systems where the eigenvalues of Hubbard states corresponding to different manifolds like $t_{2g}$ and $e_g$ are almost equal (e.g., in some high-spin compounds).

2. On the other hand, a shortcut is possible to carry out orbital-resolved DFT+$U$ calculations in a "one-step" fashion for systems whose eigenvalue ordering can be anticipated either based on previously performed calculations or from chemical intuition. In this procedure, the calculation is initialized using a starting guess of choice (e.g., superposition of atomic orbitals) and no Hubbard corrections are applied during the first few SCF iterations (typically 5-10) in order to allow the code to find a set of numerically stable eigenvectors. Subsequently, the orbital-resolved $U$ corrections are switched on and are applied to the eigenstates defined in the input file. This technique suffices to compute compounds where the occupation pattern of the involved orbitals is predictable and stabilizes quickly. For example, in low-spin $FeS_2$ or in the low-spin Fe(II) hexacomplexes, the 3rd, 4th and 5th eigenstates always correspond to the $t_g/t_{2g}$ orbitals. We have performed several tests to make sure that the one-step procedure yields the exact same ground-state as the two-step procedure.

# Influence of linear perturbations on the shape and extension of eigenstates

Table S1 shows the converged $d$-shell eigenvectors of a single Fe atom in pyrite ($FeS_2$), (1) for the unperturbed PBE ground state and (2) with a linear perturbation of 0.05 eV applied to the $t_g$ orbitals. It is immediately recognizable that the perturbation does not induce a significant change in the shape or extension of the eigenstates. This is confirmed by computing the dot products between the corresponding pairs of eigenvectors of (1) and (2), which all amount to unity up to third decimal (3). Since the eigenvectors are normalized, a dot product of one expresses that two vectors are identical in terms of both length and direction.

Table S1: Converged eigenvectors (columns) obtained from the diagonalization of the $d$-occupation matrix of a single Fe atom in pyrite ($FeS_2$), (1) for the unperturbed PBE ground state and (2) with a linear perturbation of 0.05 eV applied to the $t_g$ orbitals. (3) shows the dot product calculated between the corresponding pairs of eigenvectors from (1) and (2).

| **(1) unperturbed PBE** | | | | |
|---|---|---|---|---|
| $e_g$ eigenvectors | | $t_g$ eigenvectors | | |
| 0.172 | −0.736 | −0.652 | −0.063 | 0.000 |
| −0.281 | 0.455 | −0.601 | 0.139 | −0.577 |
| −0.253 | −0.471 | 0.421 | 0.451 | −0.577 |
| −0.736 | −0.172 | 0.063 | −0.652 | 0.000 |
| −0.534 | −0.016 | −0.180 | 0.590 | 0.577 |
| **(2) PBE+$\alpha_{\mathbf{t_{2g}}}$** | | | | |
| $e_g$ eigenvectors | | $t_g$ eigenvectors | | |
| 0.184 | −0.733 | −0.651 | −0.075 | 0.000 |
| −0.289 | 0.450 | −0.604 | 0.128 | −0.577 |
| −0.245 | −0.475 | 0.413 | 0.459 | −0.577 |
| −0.733 | −0.184 | 0.075 | −0.651 | 0.000 |
| −0.534 | −0.025 | −0.191 | 0.587 | 0.577 |
| **(3) dot products between (1) and (2)** | | | | |
| 0.99949 | 1.00001 | 1.00043 | 1.00031 | 1.00000 |

## Eigenvalue shifts of VBM and CBM due to Hubbard $U$ corrections in pyrite ($FeS_2$)

Table S2: Eigenvalues and eigenvalue shifts of VBM and CBM in pyrite ($FeS_2$) with respect to PBE ($U = 0.0\,\mathrm{eV}$).

| **setup 1 (no relaxation)** | | | | | | | | | | |
|---|---|---|---|---|---|---|---|---|---|---|
| **U (eV)** | **0.0** | **1.0** | **1.5** | **2.0** | **2.5** | **3.0** | **3.5** | **4.0** | **4.5** | **5.0** |
| VBM (eV) | 12.75 | 12.50 | 12.37 | 12.23 | 12.08 | 11.95 | 11.86 | 11.79 | 11.74 | 11.70 |
| CBM (eV) | 13.16 | 13.14 | 13.14 | 13.14 | 13.14 | 13.14 | 13.14 | 13.14 | 13.15 | 13.15 |
| $\Delta_{VBM}$ (eV) | 0.00 | −0.25 | −0.38 | −0.52 | −0.67 | −0.80 | −0.89 | −0.96 | −1.01 | −1.05 |
| $\Delta_{CBM}$ (eV) | 0.00 | −0.02 | −0.02 | −0.02 | −0.02 | −0.02 | −0.02 | −0.02 | −0.01 | 0.00 |
| **setup 2 (relaxation of atomic positions)** | | | | | | | | | | |
| **U (eV)** | **0.0** | **1.0** | **1.5** | **2.0** | **2.5** | **3.0** | **3.5** | **4.0** | **4.5** | **5.0** |
| VBM (eV) | 12.75 | 12.51 | 12.37 | 12.23 | 12.11 | 12.00 | 11.91 | 11.84 | 11.80 | 11.77 |
| CBM (eV) | 13.16 | 13.29 | 13.36 | 13.43 | 13.50 | 13.57 | 13.65 | 13.68 | 13.75 | 13.81 |
| $\Delta_{VBM}$ (eV) | 0.00 | −0.24 | −0.38 | −0.52 | −0.64 | −0.75 | −0.85 | −0.91 | −0.95 | −0.98 |
| $\Delta_{CBM}$ (eV) | 0.00 | 0.14 | 0.20 | 0.28 | 0.34 | 0.41 | 0.49 | 0.52 | 0.60 | 0.65 |
| **setup 3 (relaxation of cell vectors and atomic positions)** | | | | | | | | | | |
| **U (eV)** | **0.0** | **1.0** | **1.5** | **2.0** | **2.5** | **3.0** | **3.5** | **4.0** | **4.5** | **5.0** |
| VBM (eV) | 12.75 | 12.40 | 12.18 | 11.92 | 11.66 | 11.39 | 11.10 | 10.83 | 10.57 | 10.33 |
| CBM (eV) | 13.16 | 13.19 | 13.19 | 13.17 | 13.13 | 13.05 | 12.99 | 12.89 | 12.78 | 12.66 |
| $\Delta_{VBM}$ (eV) | 0.00 | −0.35 | −0.58 | −0.83 | −1.09 | −1.36 | −1.65 | −1.93 | −2.18 | −2.43 |
| $\Delta_{CBM}$ (eV) | 0.00 | 0.03 | 0.03 | 0.01 | −0.03 | −0.10 | −0.17 | −0.27 | −0.38 | −0.50 |

## References


(1) Anderson, E.; Bai, Z.; Bischof, C.; Blackford, S.; Demmel, J.; Dongarra, J.; Du Croz, J.; Greenbaum, A.; Hammarling, S.; McKenney, A.; Sorensen, D. *LAPACK Users' Guide*, 3rd ed.; Society for Industrial and Applied Mathematics: Philadelphia, PA, 1999.

(2) Ku, C.; Sit, P. H.-L. Oxidation-State Constrained Density Functional Theory for the

Study of Electron-Transfer Reactions. *J. Chem. Theory Comput.* **2019**, *15*, 4781–4789, PMID: 31339717.
